# Global Identification of Biofilm-Specific Proteolysis in *Candida albicans*

**DOI:** 10.1128/mBio.01514-16

**Published:** 2016-09-13

**Authors:** Michael B. Winter, Eugenia C. Salcedo, Matthew B. Lohse, Nairi Hartooni, Megha Gulati, Hiram Sanchez, Julie Takagi, Bernhard Hube, David R. Andes, Alexander D. Johnson, Charles S. Craik, Clarissa J. Nobile

**Affiliations:** a Department of Pharmaceutical Chemistry, University of California, San Francisco, California, USA; b Chemistry and Chemical Biology Graduate Program, University of California, San Francisco, California, USA; c Department of Microbiology and Immunology, University of California, San Francisco, California, USA; d Biosynesis, Inc., San Francisco, California, USA; e Tetrad Graduate Program, University of California, San Francisco, California, USA; f Department of Molecular and Cell Biology, University of California, Merced, California, USA; g Department of Medicine, University of Wisconsin, Madison, Wisconsin, USA; h Department of Medical Microbiology and Immunology, University of Wisconsin, Madison, Madison, Wisconsin, USA; i Department of Microbial Pathogenicity Mechanisms, Hans Knoell Institute, Jena, Germany

## Abstract

*Candida albicans* is a fungal species that is part of the normal human microbiota and also an opportunistic pathogen capable of causing mucosal and systemic infections. *C. albicans* cells proliferate in a planktonic (suspension) state, but they also form biofilms, organized and tightly packed communities of cells attached to a solid surface. Biofilms colonize many niches of the human body and persist on implanted medical devices, where they are a major source of new *C. albicans* infections. Here, we used an unbiased and global substrate-profiling approach to discover proteolytic activities produced specifically by *C. albicans* biofilms, compared to planktonic cells, with the goal of identifying potential biofilm-specific diagnostic markers and targets for therapeutic intervention. This activity-based profiling approach, coupled with proteomics, identified Sap5 (Candidapepsin-5) and Sap6 (Candidapepsin-6) as major biofilm-specific proteases secreted by *C. albicans*. Fluorogenic peptide substrates with selectivity for Sap5 or Sap6 confirmed that their activities are highly upregulated in *C. albicans* biofilms; we also show that these activities are upregulated in other *Candida* clade pathogens. Deletion of the *SAP5* and *SAP6* genes in *C. albicans* compromised biofilm development *in vitro* in standard biofilm assays and *in vivo* in a rat central venous catheter biofilm model. This work establishes secreted proteolysis as a promising enzymatic marker and potential therapeutic target for *Candida* biofilm formation.

## IMPORTANCE

Biofilm formation by the opportunistic fungal pathogen *C. albicans* is a major cause of life-threatening infections. This work provides a global characterization of secreted proteolytic activity produced specifically by *C. albicans* biofilms. We identify activity from the proteases Sap5 and Sap6 as highly upregulated during *C. albicans* biofilm formation and develop Sap-cleavable fluorogenic substrates that enable the detection of biofilms from *C. albicans* and also from additional pathogenic *Candida* species. Furthermore, *SAP5* and *SAP6* deletions confirm that both proteases are required for proper biofilm development *in vitro* and *in vivo*. We propose that secreted proteolysis is a promising marker for the diagnosis and potential therapeutic targeting of *Candida* biofilm-associated infections.

## INTRODUCTION

*Candida albicans* is a normal resident of the human microbiota, asymptomatically colonizing many areas of the body, including the gastrointestinal tract, genitourinary tract, oral cavity, and skin of healthy individuals. However, *C. albicans* is also the most prevalent fungal pathogen of humans. Alterations in host immunity, damaged barrier functions, stress, and resident microbiota can lead to fungal overgrowth and infections. These infections range from superficial mucosal and dermal infections, such as thrush, vaginal yeast infections, and diaper rash, to hematogenously disseminated candidiasis with mortality rates as high as 47% (1). *C. albicans* infections can be especially serious in immunocompromised individuals, such as patients undergoing chemotherapy, transplantation patients receiving immunosuppression therapy, and healthy individuals with implanted medical devices (2–4).

A major medical impact of *C. albicans* results from its ability to form resilient and drug-resistant surface-associated communities called biofilms (5–9). Biofilms can form on all implanted medical devices, including catheters, pacemakers, dentures, contact lenses, and prosthetic joints, which provide efficient substrates for biofilm growth. Biofilms can also colonize biotic surfaces, such as mucosal and epithelial cells, with life-threatening colonization and invasion of parenchymal organs occurring once cells disperse from the biofilm and enter the bloodstream, resulting in disseminated infections (5–9). In individuals with implanted medical devices, the standard treatment for device-associated biofilm infections is removal of the implanted device. This can require surgical intervention and is typically implemented after an infection has already disseminated (5–9). Therefore, the early and rapid detection of *Candida* biofilm formation is critical to improving patient outcome.

Recent genome-wide transcriptional analysis of *C. albicans* has identified certain secreted aspartyl protease (SAP)-encoding genes as being significantly upregulated during biofilm formation (10), and therefore, we hypothesized that extracellular protease activities could serve as biofilm-specific markers. To broadly test this hypothesis, we used a global approach to systematically identify extracellular protease activities in an unbiased way. To this end, a highly diverse 228-member synthetic peptide library was exposed to soluble factors produced by *C. albicans* biofilms, and the sequences cleaved in the peptide library were identified by mass spectrometry (MS). This approach, coupled with independent proteomic analyses, identified Sap5 (Candidapepsin-5) and Sap6 (Candidapepsin-6) as major biofilm-specific protease activities produced by *C. albicans*. Fluorogenic substrates that could distinguish between Sap5 and Sap6 were developed on the basis of preferential cleavages in the peptide library. These substrates both confirmed that Sap5 and Sap6 activities are highly upregulated during *C. albicans* biofilm formation and could distinguish between the biofilm and planktonic states of *C. albicans*. We also showed that these substrates could be used to detect biofilms of other pathogenic species in the *Candida* clade. To test whether Sap5 and Sap6 are required for biofilm formation, we evaluated *C. albicans* SAP5 and *SAP6* deletion strains and found that the deletions compromised biofilm formation *in vitro* on the bottom of polystyrene plates and in a microfluidic flow device, as well as *in vivo*, in a rat central venous catheter biofilm model. These results show that Sap5 and Sap6, which can be used as biofilm-specific markers, are also required for proper biofilm development.

## RESULTS

### Global protease profiling of biofilm and planktonic *C. albicans.*

**FIG 1:**
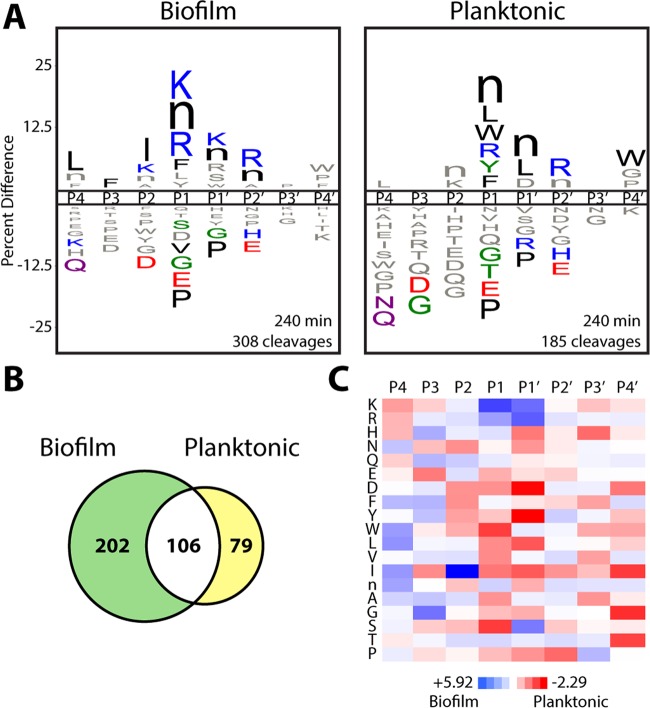
Wild-type *C. albicans* has distinct global protease substrate specificity profiles under biofilm and planktonic growth conditions. (A) iceLogo substrate specificity representations for 24-h conditioned medium from wild-type *C. albicans* (SN425) following 240 min of incubation with the MSP-MS peptide library (*P* ≤ 0.05 for residues colored by physicochemical property; “n” is norleucine). Specificity was determined with equivalent protein amounts from each condition. Specificity profiles for the 15- and 60-min assay time points are provided in Fig. S1 in the supplemental material. (B) Quantification of the total shared and unique cleavages for the biofilm and planktonic conditions at the 240-min assay time point. (C) Heat map representation of biofilm and planktonic specificity differences at the 240-min time point calculated by using *Z* score differences at the P4-P4′ positions. Biofilm-favored residues are blue (*Z* score, >0), and planktonic-favored residues are red (*Z* score, <0). A specificity comparison with time points selected to normalize for differences in proteolytic activity is provided in Fig. S2 in the supplemental material. All of the MSP-MS cleavages identified are provided in Data Set S1 in the supplemental material.

To identify global profiles of proteolytic activity associated with biofilm formation, conditioned medium from a wild-type *C. albicans* strain grown under biofilm and planktonic (suspension) conditions was assayed by a recently developed substrate-profiling approach termed Multiplex Substrate Profiling by Mass Spectrometry (MSP-MS) (11). Matched 24-h conditioned medium preparations were incubated with a physicochemically diverse library of 228 synthetic 14-mer peptide substrates (12), and time-dependent peptide cleavage products were identified through liquid chromatography-tandem MS (LC-MS/MS). Comparison of the complex cleavage profiles revealed that biofilm conditioned medium displayed an overall higher proteolytic activity against the peptide library, on the basis of the total number of unique peptide cleavage events observed throughout the assay time course (*n* = 308 for the biofilm condition and *n* = 185 for the planktonic condition at 240 min) (Fig. 1A; see Fig. S1 in the supplemental material). Motif analysis was subsequently performed to identify the global protease substrate specificities of the biofilm and planktonic conditions. iceLogo representations (13) that consider both cleaved and uncleaved positions in the peptide library were employed to visualize the fold enrichment and de-enrichment of amino acids flanking each cleavage site (cleavage occurs between the P1 and P1′ positions) (Fig. 1A; see Fig. S1). Both the biofilm and planktonic conditions shared an enrichment of bulky hydrophobic residues (e.g., norleucine) and arginine at the P1 position, hydrophobic residues at the P1′ position, and arginine at the P2′ position.

The biofilm and planktonic conditions also yielded distinct cleavages against the peptide library (*n* = 202 for the biofilm condition and *n* = 79 for the planktonic condition) (Fig. 1B). To quantify differences in global substrate specificity between these conditions, *Z* scores (13) were used to generate a difference map derived from residue preferences at each subsite (Fig. 1C). This analysis revealed significant physicochemical differences in substrate specificity. The biofilm condition displayed an increased preference for basic residues (lysine and arginine) at the P1 and P1′ positions, whereas the planktonic condition displayed an increased preference for aspartic acid at the P1′ position and certain hydrophobic residues at the P1 and P1′ positions (e.g., P1′-tyrosine). In addition, the biofilm condition displayed an overall increased preference for non-prime-side hydrophobic residues (P4-P1), with the strongest preference for isoleucine at the P2 position. Because of the higher proteolytic activity evident in the *C. albicans* biofilm conditioned medium, we also compared assay time points with similar numbers of cleavages (60 and 240 min for the biofilm and planktonic conditions, respectively) to confirm these specificity differences (see Fig. S2 in the supplemental material). Together, our substrate profiling results indicate that, in addition to exhibiting higher total activity against the peptide library, the biofilm condition is associated with a distinct protease substrate specificity profile.

### Proteomic identification of biofilm-specific proteases.

**FIG 2:**
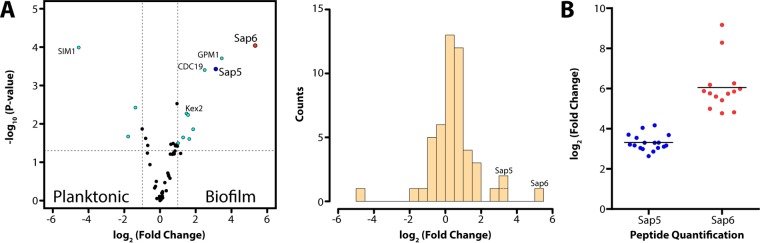
Label-free quantitation of proteins identified by shotgun proteomic analysis in conditioned medium from *C. albicans* cultures grown under biofilm and planktonic conditions. (A, left) Volcano plot depicting the log_2_-fold change in protein abundance (reported as biofilm/planktonic), demonstrating a significant increase in Sap5 and Sap6 levels under biofilm conditions with a log_2_-fold change of 3.1 for Sap5 (*P* =3.7 × 10^−4^) and a log_2_-fold change of 5.3 for Sap6 (*P* = 9.1 × 10^−5^). (A, right) Corresponding histogram showing the relative distribution of proteins identified. (B) Comparison of relative Sap5 and Sap6 peptide levels under biofilm and planktonic conditions with mean log_2_-fold changes indicated. Each data point represents quantification of a unique tryptic peptide for Sap5 or Sap6. Mean values of triplicate samples are reported. Supporting proteomic data are provided in Data Set S1 in the supplemental material.

To identify specific proteases that have increased abundance during biofilm formation, we performed shotgun proteomic analysis on matched conditioned medium preparations from wild-type *C. albicans* grown under biofilm and planktonic conditions. Two members of the Sap family, Sap5 and Sap6, were found to have significantly higher levels under biofilm conditions (8.7-fold with *P* = 3.7 × 10^−4^ for Sap5 and 40.2-fold with *P* = 9.1 × 10^−5^ for Sap6) using label-free quantitation of precursor ion abundance, with Sap5 and Sap6 being among the most differentially upregulated proteins in the biofilm state (Fig. 2). Kex2, the only other protease identified by shotgun proteomics, was found to have modestly higher abundance in biofilm conditioned medium (2.8-fold; *P* = 5.4 × 10^−3^). Kex2 is a single-pass transmembrane protease with a luminal catalytic domain that in *Saccharomyces cerevisiae* has proprotein processing roles in the *trans*-Golgi network and the late endosome/prevacuolar compartment (14). Although the release of Kex2 into conditioned medium from small amounts of nonspecific cell lysis cannot be ruled out, recent characterization of the composition of the *C. albicans* biofilm matrix identified several cytosolic metabolic proteins (15), suggesting that the extracellular localization of Kex2, possibly through extracellular vesicle release (16), may represent a relevant biofilm-specific feature.

### Biofilm-specific cleavages are attributable to Sap5 and Sap6.

**FIG 3:**
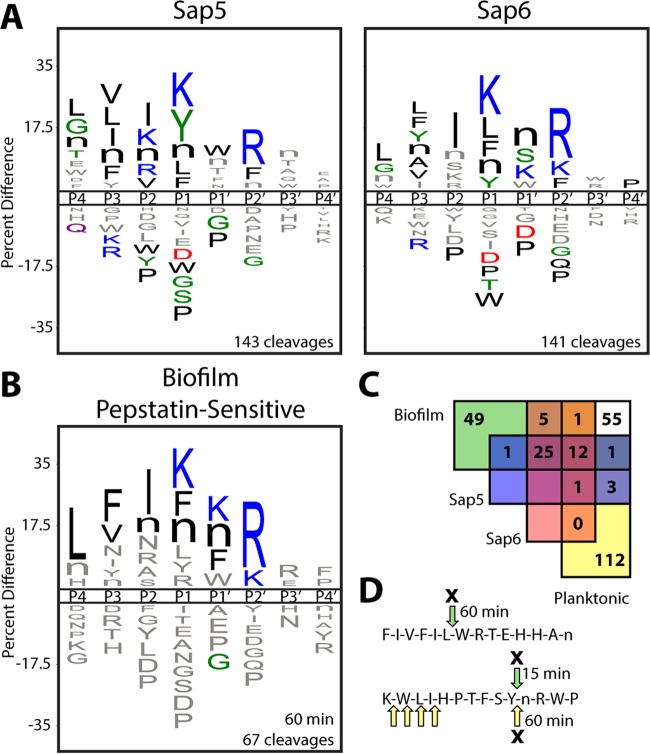
Global protease substrate specificity profiling reveals that Sap5 and Sap6 activities are highly increased in *C. albicans* under biofilm growth conditions. (A) iceLogo representations of recombinantly produced Sap5 and Sap6 following 240 min of incubation with the MSP-MS peptide library (*P* ≤ 0.05 for residues colored by physicochemical property). Further comparison of Sap5 and Sap6 specificity is shown in Fig. S4 in the supplemental material. (B) iceLogo representation of cleavages from the *C. albicans* biofilm conditioned medium MSP-MS assay that are sensitive to pretreatment with the aspartyl protease inhibitor pepstatin A (10 µM). (C) Assignment of pepstatin-sensitive cleavages in the biofilm and planktonic profiles through comparison to recombinantly produced Sap5 and Sap6. The biofilm (60 min) and planktonic (240 min) time points were chosen to normalize for the higher total proteolytic activity under the biofilm condition. iceLogo representations for unassigned cleavages are distinct from the Sap5 and Sap6 specificity profiles and are provided in Fig. S5 in the supplemental material. (D) Example peptide cleavages from the biofilm (green arrows) and planktonic (yellow arrows) MSP-MS assays are shown with pepstatin-sensitive cleavages indicated by an X and the time point of first appearance noted. Selected cleavages were omitted for clarity. All MSP-MS cleavages identified are provided in Data Set S1 in the supplemental material.

To assign biofilm-specific cleavages in the peptide library to the candidate proteases, mature Sap5, Sap6, and a soluble Kex2 construct were recombinantly expressed in *Pichia pastoris* and profiled with the MSP-MS assay. Sap5 and Sap6 were found to have broad substrate specificities against the peptide library, yielding >140 cleavages each after 240 min (Fig. 3A). Kex2 was found to have comparatively high specificity, with cleavages occurring after K/R-R (P2-P1) pairings (see Fig. S3 in the supplemental material), in agreement with its biological processing role and the specificity previously reported for recombinantly produced Kex2 proteases (17).

Assessment of the global substrate specificity profiles of recombinantly produced Sap5 and Sap6 revealed shared residue preferences. These shared preferences include lysine and bulky hydrophobic residues (phenylalanine, tyrosine, norleucine, and leucine) at the P1 position, hydrophobic residues at the P4 through P2 positions, and arginine at the P2′ position (Fig. 3A). The Sap5 and Sap6 preferences resemble the biofilm cleavage profile (Fig. 1), consistent with the upregulation of Sap5 and Sap6 under the biofilm condition. Even though mature Sap5 and Sap6 share a high degree of sequence conservation (80%) and certain residue preferences, they also produced several unique cleavages against the peptide library (30% and 29% at 240 min for Sap5 and Sap6, respectively). A difference map calculated from position-specific *Z* scores further demonstrated pronounced differences in Sap5 and Sap6 substrate specificity (see Fig. S4 in the supplemental material).

To assess the contributions of Sap5 and Sap6 specificity to the complex conditioned medium profiles, conditioned medium was pretreated with the aspartyl protease inhibitor pepstatin A prior to analysis with the MSP-MS assay. The pepstatin-sensitive cleavages under the biofilm condition demonstrated a substrate specificity motif that shared the dominant specificity features of the purified Saps and most closely resembled the Sap6 cleavage profile (Fig. 3B). Any biofilm or planktonic conditioned medium cleavages that displayed pepstatin sensitivity and matched those derived from the recombinantly produced Sap5 and Sap6 proteases were attributed to Sap5 and Sap6 activity (Fig. 3C and D). By this approach, a larger proportion of biofilm-specific (39%) than planktonic-condition-specific (3%) cleavages could be attributed to these proteases (assay time points were chosen to normalize for the higher proteolytic activity under the biofilm condition). This analysis illustrates the significant increase in Sap5 and Sap6 activity in *C. albicans* biofilms, with Sap6 accounting for a larger proportion of the biofilm-specific cleavages than Sap5.

To date, Sap family proteases are the only *C. albicans* secreted proteases that have been reported. However, our global analysis revealed cleavages not attributable to Sap5 or Sap6 from proteases that were not detectable by shotgun proteomic analysis. These unassigned biofilm-specific cleavages display an overall preference for arginine and phenylalanine at the P1 position (see Fig. S5 in the supplemental material). In contrast, the unassigned planktonic-condition-specific cleavages display a distinct preference for bulky hydrophobic residues at both the P1 and P1′ positions. Mapping of cleavage site positions along the uncapped 14-mer peptide substrates also indicated comparatively higher aminopeptidase-like activity under the planktonic condition, with the majority of unassigned cleavages occurring one to three positions from the peptide N termini (see Fig. S5).

### Construction of Sap5 and Sap6 fluorogenic peptide substrates.

**FIG 4:**
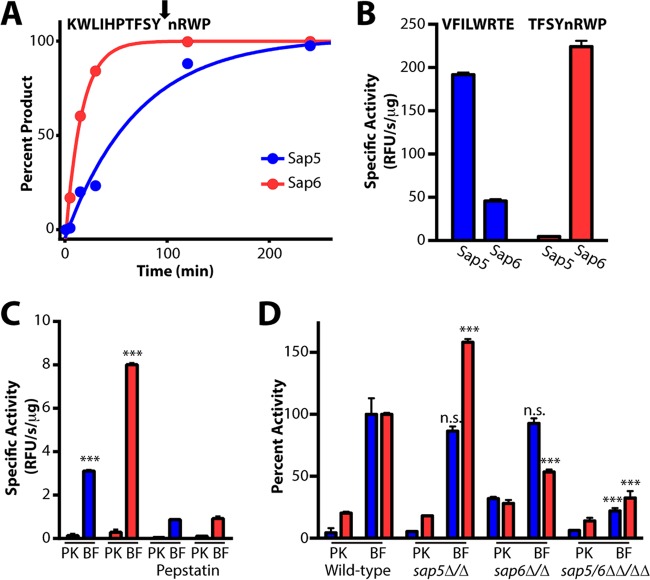
Sap5- and Sap6-cleavable fluorogenic peptide substrates distinguish between *C. albicans* under biofilm and planktonic growth conditions. (A) Fluorogenic substrates were developed on the basis of Sap5 and Sap6 cleavages from the MSP-MS peptide library. An example MS-based time course is provided showing Sap6-favored cleavage of KWLIHPTFSYnRWP, one substrate within a 25-member peptide sublibrary. Complete hydrolysis of the parent substrate and cleavage at a single site allowed for the calculation of *k*_cat_/*K_m_* values of 4.4 × 10^4^ M^−1^ s^−1^ (Sap5) and 2.0 × 10^5^ M^−1^ s^−1^ (Sap6). Cleavage time courses for the remaining peptides used in sequence selection are provided in Fig. S6 in the supplemental material. (B) Activity (relative fluorescence units [RFU] per second per microgram) of the internally quenched fluorogenic substrates VFILWRTE (blue bars) and TFSYnRWP (red bars) against recombinantly produced Sap5 and Sap6. (C) Both VFILWRTE and TFSYnRWP displayed significantly higher activity (***, *P* < 0.001) in 24-h biofilm (BF) than in planktonic (PK) conditioned medium from wild-type *C. albicans* (SN425). Pretreatment with 10 µM pepstatin A inhibits Sap5 and Sap6 activity. Similar activity and inhibition results were obtained with the wild-type SN250 reference strain and are provided in Fig. S7 in the supplemental material. (D) Cleavage of VFILWRTE and TFSYnRWP in conditioned medium from the wild-type reference (SN250) and *sap5*Δ/Δ, *sap6*Δ/Δ, and *sap5/6*ΔΔ/ΔΔ deletion strains. Deletion of both *SAP5* and *SAP6* significantly reduces the cleavage of both substrates. At least partial compensation of Sap6 activity is observed in the *sap5*Δ/Δ deletion strain. Activity was normalized to the wild-type biofilm activity for each substrate, and significant differences in biofilm activity are indicated (***, *P* < 0.001; n.s., not significant). Equivalent amounts of protein from conditioned medium preparations were used for each condition. For all results, mean activity is reported with error bars indicating the SD of triplicate values.

To develop fluorogenic probes for detecting biofilm-specific protease activity, we identified peptide sequences with selectivity for Sap5 and/or Sap6. MSP-MS peptides displaying high Sap5 and Sap6 activity were repooled into a smaller 25-member sublibrary, and a time course was used to refine cleavage preferences for these individual peptide substrates (Fig. 4A). Of the 29 cleavages with the highest activity, 8 were found to favor Sap5, whereas 13 were found to favor Sap6 (see Fig. S6 in the supplemental material). For each protease, two 8-mer peptide sequences containing P4-P4′ residues from Sap5- or Sap6-favored cleavages were selected for incorporation into internally quenched fluorogenic peptides. The fluorogenic probes were synthesized bearing either a 5-carboxyfluorescein or 7-methoxycoumarin fluorophore with a corresponding quencher positioned at the opposing terminus such that peptide cleavage yields a fluorescence signal. Among the fluorogenic substrates evaluated, the sequences VFILWRTE and TFSYnRWP were found to afford the highest selectivity for Sap5 or Sap6, respectively (Fig. 4B; see Fig. S7 in the supplemental material).

### Sap5 and Sap6 activities are upregulated under the biofilm condition.

In agreement with the prior MSP-MS cleavage site analysis, application of the fluorogenic substrates to the biofilm or planktonic conditioned medium revealed a dramatic increase in substrate cleavage under the biofilm condition, with the Sap6 substrate showing the higher induction (Fig. 4C). Pepstatin pretreatment resulted in a significant reduction in substrate cleavage, confirming predominant aspartyl protease activity (Fig. 4C). To confirm probe selectivity for the target proteases, conditioned medium preparations from *sap5*Δ/Δ and *sap6*Δ/Δ single deletion mutant strains and a *sap5/6*ΔΔ/ΔΔ double deletion mutant strain were also assayed (Fig. 4D). Deletion of both *SAP5* and *SAP6* in the double mutant significantly reduced the cleavage of both fluorogenic substrates under the biofilm condition, confirming that Sap5 and Sap6 are the major contributing activities. To our initial surprise, the individual *sap5*Δ/Δ and *sap6*Δ/Δ mutant strains did not show reductions in activity as dramatic as the *sap5/6*ΔΔ/ΔΔ double mutant strain. In the case of the individual *sap5*Δ/Δ deletion strain, activity against the Sap6 substrate increased, suggesting that upregulation of Sap6 activity is partially compensating for loss of *SAP5*. We note that the remaining substrate cleavage in the *sap5/6*ΔΔ/ΔΔ double deletion mutant strain indicates proteolytic activity from additional proteases, possibly reflecting compensation for loss of both *SAP5* and *SAP6*.

### Sap5 and Sap6 substrates enable biofilm detection across the *Candida* clade.

**FIG 5:**
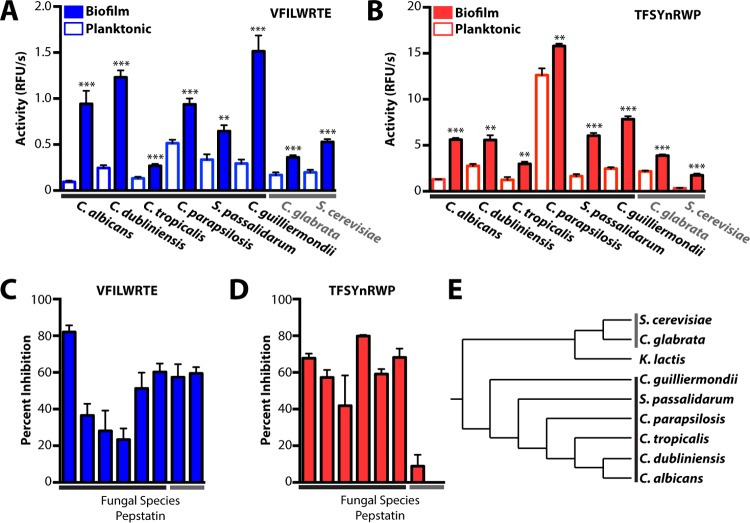
Protease activity profiling enables the detection of biofilm formation from diverse pathogenic *Candida* species. Both fluorogenic substrates VFILWRTE (blue bars) (A) and TFSYnRWP (red bars) (B) displayed significantly higher activity (relative fluorescence units [RFU] per second) (**, *P* < 0.01; ***, *P* < 0.001) in 24-h biofilm conditioned medium, indicating that proteolytic upregulation is a shared feature of biofilm formation among these fungal species. Inhibition of VFILWRTE (C) and TFSYnRWP (D) cleavage in biofilm conditioned medium following pretreatment with 10 µM pepstatin A is shown. Aspartyl protease activity against TFSYnRWP enables highly specific *Candida* clade detection. Equivalent amounts of protein from conditioned medium preparations were used for each condition. (E) Phylogenetic tree (51, 52) depicting the *Candida* clade and related fungal species. *Candida* clade species are indicated with a black bar, and the related species tested are indicated with a gray bar. For all results, mean activity is reported with error bars indicating the SD of triplicate values.

We assessed the ability of the Sap5- and Sap6-cleavable fluorogenic substrates to distinguish between the biofilm and planktonic states of diverse pathogenic species from across the *Candida* clade (Fig. 5A to E). A panel of *Candida* clade species were grown under the biofilm and planktonic conditions established for *C. albicans*, and conditioned medium preparations were assayed for proteolytic activity with the VFILWRTE and TFSYnRWP fluorogenic substrates (Fig. 5A and B). Notably, proteolytic activity against these substrates was higher under the biofilm condition than under the planktonic condition across all of the species tested, including in two species found outside the *Candida* clade. Pepstatin pretreatment of biofilm conditioned medium revealed substrate-specific differences in aspartyl protease activity. The substrate VFILWRTE exhibited a reduction in cleavage upon pepstatin treatment across all of the species tested, with *C. albicans* exhibiting the greatest pepstatin sensitivity (Fig. 5C). In contrast, the most pronounced aspartyl protease activity against the TFSYnRWP substrate was restricted to the *Candida* clade (Fig. 5D). Together, these results suggest that aspartyl protease activity is linked to conserved biofilm-associated functions and that subtle differences in proteolytic activity may distinguish between even closely related fungal species. Given the expansion of the Sap family in the *Candida* clade (18), it is possible that pepstatin-sensitive substrate cleavage within these *Candida* clade species is attributable to proteolytic activity from Sap family proteases. Although activities from different proteases or protease classes may also be relevant for cleavage of the fluorogenic substrates and even for biofilm formation in these species, proteolytic upregulation clearly distinguishes between biofilm and planktonic growth in the *Candida* clade and beyond.

### Deletion of *SAP5 and SAP6* compromises *C. albicans* biofilm formation *in vitro* and *in vivo.*

**FIG 6:**
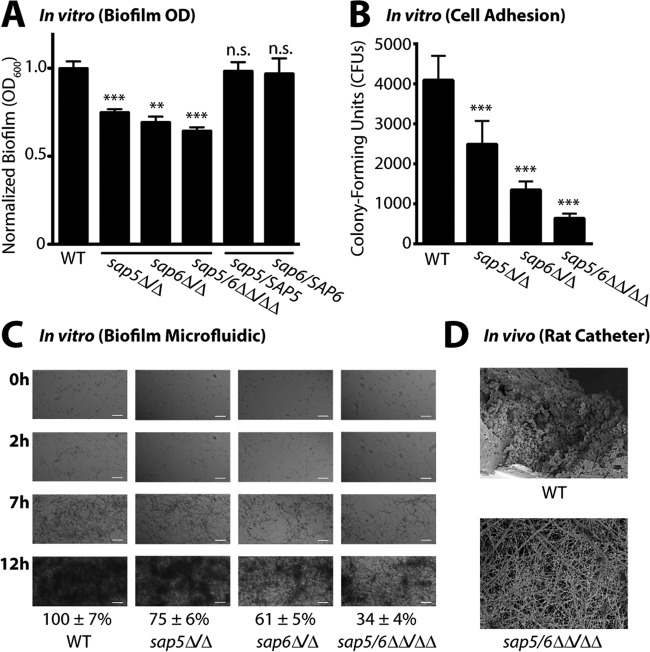
Deletion of *SAP5* and *SAP6* impairs *C. albicans* biofilm formation under *in vitro* growth conditions and *in vivo* in a rat central venous catheter biofilm model. (A) Biofilm formation in Spider medium after 24 h of growth of the wild-type (WT) reference (SN250) and *sap5*Δ/Δ, *sap6*Δ/Δ, and *sap5/6*ΔΔ/ΔΔ deletion strains. OD_600_ readings were measured for adhered biofilms after removal of the medium and normalized to the wild-type strain (OD_600_ set to 1.0), and the mean ± SD is shown (*n* = 4 for each strain). OD_600_ measurements of the *sap5*, *sap6*, and *sap5/6* deletion strains deviated significantly from those of the reference strain (**, *P* < 0.01; ***, *P* < 0.001; n.s., not significant). Complementation of the *sap5*Δ/Δ and *sap6*Δ/Δ deletion mutant strains with *SAP5* and *SAP6*, respectively, restored biofilm formation to wild-type reference levels, with *P* = 0.70 for *sap5/SAP5* and *P* = 0.66 for *sap6/SAP6* (*P* values were calculated by comparison to the reference strain). The growth rates of the *sap5* and *sap6* deletion strains in the planktonic state were not compromised, as shown in Fig. S8 in the supplemental material. (B) Quantification of early adhered cells under the biofilm assay condition in Fig. 6A immediately following the 90-min adherence period (*n* = 12 for each strain). The CFU counts (reported as mean ± SD) of the *sap5*Δ/Δ, *sap6*Δ/Δ, and *sap5/6*ΔΔ/ΔΔ deletion strains deviated significantly from those of the reference strain (***, *P* < 0.05 by one-way ANOVA). (C) Time-dependent visualization of biofilm formation under dynamic flow (0.5 dyne/cm^2^) in Spider medium over a 12-h period postadherence with a BioFlux 1000z instrument, with representative 0-, 2-, 7-, and 12-h images shown (*n* = 3 for each strain). Bars = 50 µm in all panels. Areas occupied by the mature biofilms at 12 h were normalized to the wild-type (SN250) reference strain. Normalized percent areas (reported as mean ± SD) of the *sap5*Δ/Δ(75 ± 6%), *sap6*Δ/Δ(61 ± 5%), and *sap5*/*6*ΔΔ/ΔΔ (34 ± 4%) deletion strains deviated significantly (*P* < 0.01) from those of the reference strain (100 ± 7%). Corresponding time-lapse videos of biofilm formation are provided in Movie S1 in the supplemental material. (D) Comparison of *in vivo* biofilm formation by the *C. albicans* wild-type reference (SN250) and *sap5/6*ΔΔ/ΔΔ deletion strains in the rat central venous catheter biofilm model. SEM images acquired at 24 h postadherence illustrate a reduction in the number of adhered cells of the *C. albicans* sap5/6ΔΔ/ΔΔ deletion strain (host protein deposited on the intraluminal surface is evident). Representative images of biological duplicates are shown at ×1,000 magnification. White bars, 10 µm. The *sap5/6*ΔΔ/ΔΔ deletion did not affect planktonic virulence *in vivo* as shown in Fig. S8 in the supplemental material.

We next evaluated the effects of *SAP5*, *SAP6*, and *SAP5/6* deletions on *C. albicans* biofilm formation *in vitro* and *in vivo*. Biofilms were quantified with a standard biofilm assay using optical density at 600 nm (OD_600_) after 24 h of growth in a multiwell plate format as described previously (19). This analysis identified a significant reduction in biofilm formation by the *sap5*Δ/Δ, *sap6*Δ/Δ, and *sap5/6*ΔΔ/ΔΔ deletion strains compared to that by the wild-type reference strain, with the *sap6*Δ/Δ and *sap5/6*ΔΔ/ΔΔ mutants having the most noticeable defects (Fig. 6A). Reintroduction of wild-type alleles of *SAP5* and *SAP6* into the *sap5*Δ/Δ and *sap6*Δ/Δ deletion mutant strains, respectively, restored normal biofilm levels, validating the functional roles of both *SAP5* and *SAP6* in biofilm formation *in vitro*. The wild-type and mutant strains all displayed comparable growth rates in planktonic cultures (see Fig. S8 in the supplemental material), indicating that the biofilm defects were not a result of inherent growth differences.

Because of the reduction in *C. albicans* biofilm formation, we independently quantified cellular adhesion under early biofilm-forming conditions immediately following the initial (90-min) adherence period. This analysis revealed a highly pronounced reduction in the number of adhered cells of the *sap5*Δ/Δ, *sap6*Δ/Δ, and *sap5/6*ΔΔ/ΔΔ deletion mutant strains relative to those of the wild-type strain (Fig. 6B) that correlated with their biofilm defects. The *sap5/6*ΔΔ/ΔΔ deletion strain exhibited an 85% reduction compared to the wild-type reference, suggesting that *SAP5* and *SAP6* play essential roles in initiating proper biofilm formation *in vitro* by contributing to cellular adhesion during the first stage of biofilm formation.

To better mimic physiological growth conditions, time-dependent biofilm formation for the *sap5*Δ/Δ, *sap6*Δ/Δ, and *sap5/6*ΔΔ/ΔΔ mutant and wild-type reference strains was also visualized in a microfluidic assay under dynamic flow conditions equivalent to the average flow rate of fluid across a typical intravascular human catheter (Fig. 6C) (20–22). Time-lapse microscopy videos of biofilm development were recorded for 12 h postadherence to the microfluidic chamber surface (see Movie S1 in the supplemental material). In agreement with the OD_600_ measurements, quantification of biofilm formation under dynamic flow conditions revealed a marked reduction in biofilm growth for the *sap6*Δ/Δ and *sap5/6*ΔΔ/ΔΔ mutant strains compared to the wild-type reference strain and a less pronounced defect for the *sap5*Δ/Δ strain (Fig. 6C). This assay suggests that *SAP5* and *SAP6* play roles in *C. albicans* biofilm formation over time, with *SAP6* playing the larger role as the biofilm matures.

Finally, we evaluated the effect of *SAP5/6* deletion on *in vivo C. albicans* biofilm formation with a rat central venous catheter biofilm model (23). Catheters surgically implanted in the jugular vein underwent intraluminal infection with the *sap5/6*ΔΔ/ΔΔ mutant strain or the wild-type reference strain. Following a 4-h adherence period, the catheters were flushed, and after 24 h, biofilm formation on the intraluminal catheter surface was assessed by scanning electron microscopy (SEM). The *sap5/6*ΔΔ/ΔΔ deletion strain exhibited a significant reduction in biofilm formation *in vivo* compared to the wild-type reference strain, as noted by a visibly evident decrease in *C. albicans* cells that had adhered to the catheter surface (Fig. 6D). The lack of significant biofilm formation *in vivo* in the rat catheter model and in the *in vitro* biofilm models suggests that Sap5 and Sap6 play key roles in the formation of device-associated biofilms and that this may occur, at least in part, by contributing to cellular adhesion during biofilm initiation.

To evaluate the effect of *SAP5/6* deletion on *C. albicans* planktonic virulence *in vivo*, we also tested the *sap5/6*ΔΔ/ΔΔ mutant strain in a murine tail vein injection model of hematogenously disseminated candidiasis (which does not require biofilm formation). In agreement with a recent report (24), the *sap5/6*ΔΔ/ΔΔ deletion mutant strain did not show a reduction in *C. albicans* planktonic virulence compared to the wild-type reference strain in intravenously infected BALB/c mice on the basis of both survival time and kidney burden (see Fig. S8 in the supplemental material). This result is consistent with our observation that deletion of *SAP5* and *SAP6* does not compromise the growth of *C. albicans* in the planktonic state but that it has a profound effect on biofilm formation.

## DISCUSSION

The ability of *Candida* species to form biofilms on biotic surfaces and implanted medical devices provides a major source of new infections and presents a special treatment challenge, as biofilms are resistant to conventional antifungal drugs (8). Early and rapid diagnosis of biofilm-associated infections is necessary to improve patient outcomes and requires the identification of biofilm-specific molecular markers. Beginning with a global activity-based approach, we identified Sap5 and Sap6 as major secreted biofilm-specific protease activities. Global substrate specificity profiling, coupled with the design of specific peptide substrates, showed that Sap5 and Sap6 activities greatly increase during *C. albicans* biofilm formation and that our fluorogenic probes could distinguish between the biofilm and planktonic states of *C. albicans*. We also showed that this is true for several additional pathogenic species in the *Candida* clade. Finally, the deletions of *SAP5* and *SAP6* in *C. albicans* demonstrated that these proteases are essential for proper biofilm formation both *in vitro* and *in vivo*.

Previous efforts to characterize the functions of Sap5 and Sap6 have focused on their potential invasive roles (25–28). Indeed, *SAP5* and *SAP6* have been shown to be hyphae-specific genes (29, 30), with *SAP6* induction observed in hyphae-infiltrated tissue (27). However, the results described here suggest that Sap5 and Sap6 have functions during biofilm formation distinct from the hyphal invasion of host tissues. Defects in cellular adhesion in the *SAP5* and *SAP6* deletion strains demonstrate that these proteases are required for proper adhesion by *C. albicans* during the initiation of biofilm formation. Sap5- and Sap6-mediated adhesion of *C. albicans* cells to abiotic surfaces may help to both seed the formation of biofilms and maintain their attachment to catheters and other implanted medical devices. In addition, *SAP5* and *SAP6* expression is dynamic during biofilm development (19, 31), suggesting that they may play a variety of different roles throughout biofilm maturation. For example, Sap5 and Sap6 may contribute to nutrient breakdown and acquisition, cell-cell communication, or extracellular matrix production. Indeed, expression of *SAP5* and *SAP6* is considerably higher (>50-fold) *in vivo* in mature biofilms isolated from the rat catheter model than in planktonic cells (31), further supporting the relevance of Sap5 and Sap6 in mature biofilm infections.

The strong induction of Sap5 and Sap6 proteolytic activities in biofilms and *in vivo* upregulation suggests that Sap5 and Sap6 may be promising biofilm diagnostic markers. Current clinical approaches for diagnosing *Candida* infections are hampered by low sensitivity, slow turnaround times, and a lack of species specificity (32, 33). These approaches also fail to distinguish between biofilm and planktonic cells. We envision that fluorogenic peptide substrates with biofilm (or planktonic) selectivity could be used as *ex vivo* diagnostic tools through noninvasive blood sampling. The catalytic signal amplification by proteolytic activity (34) makes these probes particularly sensitive. Development of rapid and noninvasive approaches for detecting *Candida* biofilm formation would change current clinical practices by enabling the early identification of biofilms, well before they have had a chance to seed life-threatening disseminated infections.

Although the main goal of our work is to ultimately discover rapid diagnostics for *C. albicans* biofilms, we also found that Sap5 and Sap6 are necessary for biofilm formation *in vitro* and *in vivo*, suggesting that they may be useful as biofilm-specific therapeutic targets for preventing or inhibiting biofilm growth. Saps produced by *C. albicans* during infections have been previously suggested as therapeutic targets. For example, HIV aspartyl protease inhibitors were observed to inhibit *C. albicans* growth in the clinic, possibly by inhibiting various Saps (35, 36). In all, *C. albicans* has 10 *SAP* genes, and they have previously been implicated in many aspects of pathogenesis, including invasion, cell wall protein shedding, hyphal cell aggregation, nutrient acquisition, activation of the host inflammatory response, and immune escape (25, 37–40). Although the precise mechanistic roles of Sap5 and Sap6 in biofilm formation are not yet known, the substrate specificity preferences identified here may ultimately lead to the discovery of their endogenous substrates.

## MATERIALS AND METHODS

### Reagents.

The reagents used in this study were obtained from the following commercial sources. BD Bacto peptone, BD Bacto yeast extract, BD Difco yeast nitrogen base without amino acids, and BD Difco nutrient broth were from Becton, Dickinson, and Company. RPMI 1640 medium with l-glutamine and 4-morpholinepropanesulfonic acid (MOPS) and without sodium bicarbonate was from Lonza. Phosphate-buffered saline (PBS), Dulbecco’s PBS (D-PBS), ampicillin, and gentamicin were from the University of California, San Francisco (UCSF) cell culture facility. Glucose and acetonitrile were from Fisher. Biotin, methanol, mono- and dibasic potassium phosphate, citric acid, sodium chloride, 2-[bis(2-hydroxyethyl)amino]-2-(hydroxymethyl)propane-1,3-diol (BIS-Tris), dithiothreitol (DTT), iodoacetamide, ammonium bicarbonate, pepstatin A, EDTA, 2-(*N*-morpholino)ethanesulfonic acid (MES), *N*,*N*-dimethylformamide (DMF), *N*-methylmorpholine, 4-methylpiperidine, triisopropylsilane, Triton X-100, and mannitol were from Sigma-Aldrich. Glycerol was from Acros Organics. Urea, formic acid, and potassium chloride were from J. T. Baker. Trifluoroacetic acid was from Thermo Scientific. Peptide synthesis reagents were from AnaSpec, Inc., or Novabiochem (EMD Millipore) unless otherwise noted.

### Strains.

The wild-type *C. albicans* SN425 and SN250 strains (SC5314 background) have been described previously (41). Construction of the *sap5*Δ/Δ (SAP5MS4B) and *sap6*Δ/Δ (SAP6MS4B) single and *sap5/6*ΔΔ/ΔΔ (SAP56MS4B) double mutant strains has also been described previously (42). *SAP5* and *SAP6* complementation strains CJN2831 and CJN2833, in the *sap5*Δ/Δ and *sap6*Δ/Δ mutant backgrounds, respectively, were constructed by cloning *SAP5* and *SAP6* exons (containing the 800-bp sequence upstream of the start codon and the 500-bp sequence downstream of the stop codon) into plasmid pJCP055 (43); integration into *C. albicans* was verified by colony PCR. For *C. dubliniensis* (CD36), *C. tropicalis* (ATCC MYA-3404), *Spathaspora passalidarum* (ATCC MYA-4345/NRRL Y-27907), and *C. glabrata* (CBS138), a sequenced strain was used; isolates other than the sequenced strain were used for *C. parapsilosis* (ATCC 22019/CBS604) and *Meyerozyma/C. guilliermondii* (ATCC 9058/CBS2021). For *S. cerevisiae*, we used EM93, a precursor of the sequenced S288c strain that is capable of flocculation (44).

### Conditioned medium preparation.

*C. albicans* strains were grown overnight at 30°C in yeast extract-peptone-dextrose (YPD) medium. These cultures were diluted to an OD_600_ of 0.5 or 0.05 in RPMI medium for the biofilm and planktonic conditions, respectively, and grown under filamentation conditions. For planktonic cultures, two 150-ml Erlenmeyer flasks with 25 ml of RPMI medium were seeded with overnight culture and grown for 24 h at 37°C in a New Brunswick Scientific incubator while shaking at 300 rpm. *C. albicans* biofilm cultures were grown in six-well non-tissue-culture polystyrene plates by first seeding 4 ml of RPMI medium with an overnight culture and allowing cells to adhere for 90 min at 200 rpm in an ELMI plate shaker set to 37°C. Nonadherent cells were washed with 4 ml of PBS, 4 ml of fresh RPMI medium was added, and biofilms were grown for 24 h. Conditioned medium was harvested by collecting both *C. albicans* cells and conditioned medium from two flasks of planktonic culture or two plates of biofilm culture and spinning it at 3,750 rpm for 10 min to pellet the cells. This supernatant was pooled and then filtered with a 0.45-µm syringe filter prior to being flash frozen in liquid nitrogen and stored at −80°C. Thawed conditioned medium was concentrated through centrifugation with an Amicon Ultra spin filter with a 10-kDa cutoff (EMD Millipore) and buffer exchanged through >10-fold dilution into D-PBS (pH 7.4) prior to a final spin concentration step. Protein was quantified by the method of Bradford and stored at −80°C.

Conditioned medium from other species (and for *C. albicans* conditioned medium being compared to these other species) was harvested as described above, with the following modifications. RPMI medium was supplemented with 2% glucose for both planktonic and biofilm growth conditions. Conditioned medium was harvested by collecting four flasks of planktonic culture or four plates of biofilm culture. Supernatants from the different flasks or plates for a given strain were pooled following the D-PBS spin concentration step. We note that the medium condition used (RPMI medium with 2% glucose) was chosen because of robust *C. albicans* biofilm formation and may not be optimal for other species.

### Recombinant protein expression.

Recombinant Sap5, Sap6, and Kex2 were produced with a *P. pastoris* expression system using strains that have been described previously (45, 46). Kex2 from *S. cerevisiae* was selected for the present study because it has been produced successfully in *P. pastoris*, unlike *C. albicans* Kex2, and has similar substrate specificity (17). *P. pastoris* cultures (1 liter) were grown to saturation in buffered glycerol complex medium for 2 days at 30°C. Cells were harvested and resuspended in 200 ml of buffered methanol complex medium containing 0.5% methanol. Protein expression was carried out for 2 days for Sap5 and Sap6 and for 1 day for Kex2. Culture supernatant was passed through a 0.22-µm vacuum filter and stored at −80°C.

### Recombinant protein purification.

Recombinant *C. albicans* Sap5 and Sap6 were purified by an adaptation of the methods of Borelli et al. (47). Briefly, culture supernatant was concentrated to 15 ml with a spin filter with a 10-kDa cutoff and dialyzed against 4 liters of 10 mM sodium citrate buffer (pH 7.0) for 4 h, followed by a second overnight dialysis. Dialyzed supernatant was concentrated to 5 ml with a spin filter with a 10-kDa cutoff and loaded at 0.5 ml/min onto a 5-ml HiTrap SP HP column (GE Healthcare) that had been equilibrated with 10 mM sodium citrate (pH 7.0). Protein was resolved at 4 ml/min with a gradient of 10 mM sodium citrate (pH 7.0) containing 300 mM NaCl while 5-ml fractions were collected. Fractions containing the highest-purity Sap5 or Sap6 as assessed by SDS-PAGE were pooled and concentrated to 1 ml with a spin filter with a 10-kDa cutoff. Pooled protein was loaded at 0.25 ml/min onto a Superdex 200 10/300-GL column (GE Healthcare) that had been equilibrated with 10 mM sodium citrate (pH 7.0) containing 150 mM NaCl. Protein was resolved with an isocratic flow of 1 ml/min while 1-ml fractions were collected. Fractions containing purified Sap5 or Sap6, as assessed by SDS-PAGE, were pooled and concentrated to 1 ml.

For recombinant *S. cerevisiae* Kex2, culture supernatant was prepared as described above for Sap5 and Sap6. However, dialysis was performed with 50 mM BIS-Tris (pH 5.0). Concentrated supernatant was loaded at 0.5 ml/min onto a 5-ml HiTrap Q HP column (GE Healthcare) that had been equilibrated with 50 mM BIS-Tris (pH 5.0). Protein was resolved at 4 ml/min with a gradient of 50 mM BIS-Tris (pH 5.0) containing 1 M NaCl while 5-ml fractions were collected. Kex2-containing fractions were pooled and further purified by size exclusion chromatography as described above, except employing an isocratic flow of 50 mM BIS-Tris (pH 5.0) containing 150 mM NaCl. Pooled fractions were exchanged into 50 mM BIS-Tris (pH 7.2) containing 50% glycerol with a PD-10 desalting column (GE Healthcare) prior to concentration. All recombinant proteins were quantified with *A*_280_ molar extinction coefficients calculated from the Expasy ProtParam tool and stored at −80°C.

### Proteomic analysis of *C. albicans* biofilm and planktonic condition medium.

Triplicate 24-h conditioned medium preparations (4 µg) from matched wild-type *C. albicans* (SN425) cultures grown under biofilm and planktonic conditions were incubated with 6 M urea and 10 mM DTT for 20 min at 55°C. Samples underwent alkylation with 12.5 mM iodoacetamide in the dark at ambient temperature for 1 h. Samples were quenched with 10 mM DTT, and the final volume was diluted 3-fold into 25 mM ammonium bicarbonate. Trypsin digestion was performed with a 1:20 ratio of sequencing-grade trypsin (Promega) to total protein overnight at 37°C. Samples were acidified to approximately pH 2 with formic acid. Peptides were desalted with C_18_ Desalting Tips (Rainin), lyophilized, and rehydrated in 0.2% formic acid.

Peptide sequencing by LC-MS/MS was performed on an LTQ-Orbitrap XL mass spectrometer (Thermo) equipped with a nanoACQUITY (Waters) ultraperformance LC (UPLC) system and an EASY-Spray ion source (Thermo). Reversed-phase chromatography was carried out with an EASY-Spray PepMap C_18_ column (Thermo, ES800; 3-µm bead size, 75 µm by 150 mm). The LC was operated at a 600-nl/min flow rate during sample loading for 20 min, the flow rate was reduced to 300 nl/min, and then peptides were separated over 30 min with a linear gradient of 2 to 50% (vol/vol) acetonitrile in 0.1% formic acid. For MS/MS analysis, survey scans were recorded over a mass range of 325 to 1,500 *m/z*. Peptide fragmentation was performed by collision-induced dissociation (CID) on the six most intense precursor ions, with a minimum of 1,000 counts, with an isolation width of 2.0 *m/z* and a minimum normalized collision energy of 25. Internal recalibration to polydimethylcyclosiloxane ion (*m/z* =445.120025) was used for both MS and MS/MS scans.

MS peak lists were generated with in-house software called PAVA. Database searching was performed with Protein Prospector software, v.5.16.1 (http://prospector.ucsf.edu/prospector/mshome.htm, UCSF) (48) against the UniProtKB *C. albicans* (SC5314, taxonomic identifier 237561) database (downloaded 17 June 2013; 9,111 entries). The database was concatenated with an equal number of fully randomized entries for estimation of the false-discovery rate (FDR). Database searching was carried out with tolerances of 20 ppm for parent ions and 0.8 Da for fragment ions. Peptide sequences were matched as tryptic peptides with up to two missed cleavages. Constant and variable modifications were set as described previously (11). The following Protein Prospector score thresholds were selected to yield a maximum protein FDR of less than 1%. A minimum “protein score” of 22 and a minimum “peptide score” of 15 were used; maximum expectation values of 0.01 for protein and 0.05 for peptide matches were used. Proteins with a minimum of two unique peptides for identification are reported. MS1 extracted ion chromatograms for label-free quantitation were obtained from Skyline software (v.3.5; University of Washington) (49). Statistical significance (*P* values) was calculated with a two-tailed *t* test. Supporting search and quantitation results are provided (see Data Set S1 in the supplemental material) for all of the proteins identified in matched biofilm and planktonic conditioned media from the wild-type (SN425) strain.

### Multiplex substrate profiling by MS (MSP-MS).

Substrate specificity profiles were determined for 24-h conditioned medium from wild-type *C. albicans* (SN425) biofilm and planktonic cultures and for recombinant Sap5, Sap6, and Kex2 with the MSP-MS assay (11). Conditioned medium preparations were profiled in the absence of inhibitor and following 30 min of preincubation on ice with 10 µM pepstatin A or 1 mM EDTA. Recombinant Saps and Kex2 were profiled in an identical manner in the absence of inhibitor and following pretreatment with either 10 µM pepstatin A (Saps) or 1 mM EDTA (Kex2). MSP-MS assays were carried out as described previously (11). Briefly, 20 µg/ml conditioned medium, 2 µg/ml Sap5, 0.2 µg/ml Sap6, 2 µg/ml Kex2, and matched no-enzyme controls were assayed against a diverse library of 228 tetradecapeptides pooled at 500 nM in D-PBS (pH 7.4) for Kex2 or matched MES at pH 5.5 (9.5 mM MES, 2.7 mM KCl, 140 mM NaCl) for recombinant Saps and conditioned medium. Sap5 and Sap6 concentrations were selected to normalize for cleavage number. After 15, 60, and 240 min, 30 µl of assay mixture was removed, quenched with 7.5 µl of 20% formic acid, and flash-frozen in liquid N_2_. For sublibrary profiling with the recombinant Saps, 0.2 µg/ml Sap5 and Sap6 were assayed in the MES buffer (pH 5.5) against a sublibrary of 25 tetradecapeptides pooled at 500 nM. After 1, 5, 15, 30, 120, 240, and 1,440 min, 29 µl of the assay mixture was removed and quenched with a mixture of 7.5 µl of 20% formic acid and 1 µl of 375 µM pepstatin A prior to being flash frozen in liquid N_2_. All peptide samples were desalted with C_18_ Desalting Tips, lyophilized, and rehydrated in 0.2% formic acid.

Cleavage site identification was performed with the LTQ Orbitrap-XL mass spectrometer, ion source and UPLC system described above. LC was done at a 600-nl/min flow rate during sample loading for 14 min and then at a 300-nl/min flow rate for peptide separation over 65 min with a linear gradient of 2 to 50% (vol/vol) acetonitrile in 0.1% formic acid. Peptide fragmentation was performed with the CID parameters described above. MS peak lists were generated with MSConvert, and data were searched against the 228-member peptide library with Protein Prospector and tolerances of 20 ppm for parent ions and 0.8 Da for fragment ions. All cleavages were allowed in the search by designating no enzyme specificity. The following variable modifications were used: amino acid (proline, tryptophan, and tyrosine) oxidation and N-terminal pyroglutamate conversion from glutamine. Protein Prospector score thresholds were selected with a minimum protein score of 22 and a minimum peptide score of 15. For the peptide sublibrary, a minimum protein score of 15 and a minimum peptide score of 10 were used. Maximum expectation values of 0.01 and 0.05 were selected for protein and peptide matches, respectively. Peptides corresponding to cleavage products in the 228-member library (12) were imported into iceLogo software v.1.2 (13) to generate substrate specificity profiles as described previously (11). Octapeptides corresponding to P4-P4′ were used as the positive data set, and octapeptides corresponding to all possible cleavages in the library (*n* = 2,964) were used as the negative data set. Octapeptide cleavage products identified by the MSP-MS assays with the 228-member library are provided in Data Set S1 in the supplemental material. Kinetic calculations for the peptide sublibrary were performed as described previously (11) except by employing precursor ion intensities for progress curve calculations. Data fitting was performed with Prism (v.6.0).

### Peptide synthesis of fluorogenic substrates.

The internally quenched fluorogenic substrate VFILWRTE, bearing a 5-carboxyfluorescein (5-FAM) fluorophore and a paired CPQ2 quencher as CPQ2–Val–Phe–Ile–Leu–Trp–Arg–Thr–Glu–Lys(5-FAM)–d-Arg–d-Arg–NH_2_, was custom synthesized by CPC Scientific, Inc. The remaining substrates (IYRnHVQL, WPSnNKVG, and TFSYnRWP) were synthesized in house with a 7-methoxycoumarin (MCA) fluorophore and a 2,4-dinitrophenyl (DNP) quencher by standard 9-fluorenylmethoxycarbonyl (Fmoc) peptide synthesis chemistry. The full peptide sequences are d-Arg–d-Arg–Lys(MCA)–Ile–Tyr–Arg–Nle–His–Val–Gln–Leu–Lys(DNP), d-Arg–d-Arg–Lys(MCA)–Trp–Pro–Ser–Nle–Asn–Lys–Val–Gly–Lys(DNP), and d-Arg–d-Arg–Lys(MCA)–Thr–Phe–Ser–Tyr–Nle–Arg–Trp–Pro–Lys(DNP). The first eight Fmoc-protected amino acids of each peptide were coupled to 150 mg of preloaded Fmoc-Lys(DNP) Wang resin (AnaSpec, Inc.) with a Symphony Quartet 4-channel peptide synthesizer (Protein Technologies, Inc.). Double couplings for all steps were performed in DMF with Fmoc-amino acid (6.5 eq), *N*-methylmorpholine (13 eq), and 2-(1H-benzotriazol-1-yl)-1,1,3,3-tetramethyluronium hexaﬂuorophosphate (HBTU) (6.5 eq), with the following exception. The Fmoc-Lys(MCA)-OH (AnaSpec, Inc.) fluorophore (3 eq) underwent a single overnight coupling with *N*-methylmorpholine (6 eq) and HBTU (3 eq). Fmoc deprotection for each step was afforded with 20% (vol/vol) 4-methylpiperidine in DMF. Trifluoroacetic acid (TFA) cleavage was carried out with a solution of TFA (95%, vol/vol), water (2.5%, vol/vol), and triisopropylsilane (2.5%, vol/vol). Peptides were precipitated in diethyl ether, and the crude material was dried under ambient conditions. Peptides were purified on a preparative Vydac C_18_ column (22 by 250 mm, 10 µm) by reversed-phase high-performance LC (HPLC) with a gradient of 95% acetonitrile in 0.1% aqueous TFA. Matrix-assisted laser desorption ionization (MALDI) mass spectra were recorded on an Applied Biosystems Voyager DE-STR MALDI time-of-flight apparatus in positive-ion mode with a 1:1 (vol/vol) α-cyano-4-hydroxycinnamic acid-to-sample ratio. MS calculated (found): (M + H) IYRnHVQL, 1,991.0 (1,993.0); (M + H) WPSnNKVG, 1,849.9 (1,851.6); TFSYnRWP (M + H), 2,019.0 (2,020.6).

### Protease activity assays.

Activity assays were performed for up to 1 h in black 96-well round-bottom plates (Costar) with a BioTek Synergy H4 hybrid multimode microplate reader set to 37°C. The excitation and emission wavelengths used were, respectively, 328 and 393 nm (gain, 96) for MCA and 485 and 538 nm (gain, 94) for 5-FAM. Activity assays with conditioned medium were performed with 10 µM substrate and 10 or 20 µg/ml protein sample for VFILWRTE and 10 µg/ml protein sample for TFSYnRWP. For assays with recombinant Saps, 2 µg/ml Sap5 and Sap6 were used with the following substrate concentrations: VFILWRTE, 10 µM; IYRnHVQL, 25 µM; WPSnNKVG, 25 µM; TFSYnRWP, 10 µM. Preincubation with 10 µM pepstatin A was performed for 30 min on ice. All activity assays were performed in the MES (pH 5.5) buffer described above, which contained 0.01% Triton X-100 for recombinant enzyme activity measurements. Initial rates from a linear fit of the progress curves obtained with Gen5 Software v.2.03 are reported.

### *In vitro* biofilm formation phenotype assays.

Biofilm formation assays using OD_600_ measurements were carried out as described previously (19). For adhesion assays, cells were allowed to adhere for 90 min in 96-well non-tissue-culture-treated polystyrene plates (Falcon) under biofilm-forming conditions. Nonadhering cells were subsequently removed, and the wells were washed twice with 200 µl of PBS. Adhering cells were vigorously resuspended in sterile water, serial dilutions were made, dilutions were plated onto YPD plates for 2 days at 30°C, and cells were quantified on the basis of CFU counting. Statistical significance was determined by one-way analysis of variance (ANOVA) (*n* = 12 for each biological sample). Time-dependent biofilm formation assays were performed under flow with a BioFlux EZ1000 (Fluxion Biosciences) microfluidic instrument. Briefly, overnight cultures grown at 30°C in YPD medium were diluted to a final OD_600_ of 0.5 in Spider medium. Cells for each strain were seeded in three replicates on a BioFlux 48-Well Low-Shear plate (Fluxion Biosciences) and allowed to adhere at 37°C for 20 min. Nonadhering cells were washed away with flowing medium at 1 dyne/cm^2^ for 5 min. The biofilms were grown for 12 h at 37°C with medium passage at 0.5 dyne/cm^2^, and time-lapse images were captured every 5 min with a Zeiss AX10 microscope with a 20× objective for bright-field and phase-contrast microscopy for each well. Three fields of vision were used for each replicate, and representative images from select time points are shown. The mature biofilm was quantified with the BioFlux Montage software (Fluxion Biosciences), and the data are reported as percentages of the area occupied by the mature wild-type (SN250) biofilm, which was assigned a value of 100%. For representative videos of each strain, see Movie S1 in the supplemental material.

### *In vitro* planktonic growth assays.

Growth assays were performed by previously described methods (50). Briefly, cells from an overnight culture grown in YPD medium at 30°C were inoculated into 100 µl of YPD medium at a starting OD_600_ of 0.01. The assay was performed with six replicates of each strain in flat-bottom 96-well plates (BD Falcon). Growth curves were generated in a BioTek plate reader at 30°C with orbital shaking at 400 rpm. OD_600_ measurements were taken every 15 min for 24 h.

### Animal models of *C. albicans* infection.

All of the vertebrate animal experiments performed in this study were approved by the Institutional Animal Care and Use Committee at the appropriate institution that participated in the research. The rat central venous catheter biofilm model was used as previously reported (19, 23). Briefly, pathogen-free male rats (Sprague-Dawley) were implanted with a heparinized (100 U/ml) polyethylene catheter (0.76-mm inside diameter, 1.52-mm outside diameter), which was inserted into the external jugular vein and advanced to a site above the atrium. Catheters were inserted 24 h prior to infection to permit deposition of host protein. Infection was achieved through intraluminal instillation with 500 µl of the *sap5/6*ΔΔ/ΔΔ deletion mutant or SN250 wild-type reference strain (10^6^ cells/ml) with two rats per strain. The catheter volume was withdrawn and flushed with heparinized 0.15 M NaCl after a 4-h dwelling period, and catheters were removed after 24 h to assess biofilm growth on the intraluminal surface by SEM.

For the mouse model of hematogenously disseminated candidiasis (24), 12 female BALB/c mice (Simonsen Laboratories) weighing between 18 and 20 g were infected with the *sap5/6*ΔΔ/ΔΔ deletion mutant or SN250 wild-type reference strain. Saturated *C. albicans* cultures were grown overnight in YPD medium at 30°C for 12 h. Cells were washed twice with sterile saline, and either 5.3 × 10^5^ cells (wild-type SN250) or 5.4 × 10^5^ cells (*sap5/6*ΔΔ/ΔΔ deletion mutant) in a 0.1-ml volume were delivered via tail vein injection. Mice were monitored daily and sacrificed when moribund. Three mice were injected with saline solution as a noninfected control. For kidney burden measurements, kidneys were dissected and flash frozen at the time of sacrifice. Kidneys were washed, placed in 1 ml of saline solution, and homogenized. Homogenates were plated onto synthetic complete medium containing ampicillin (50 µg/ml) and gentamicin (15 µg/ml) for CFU counting.

### Statistics.

Statistical significance (*P* values) was calculated with a two-tailed *t* test, except where noted otherwise. For protease activity assays with fluorogenic substrates, mean activity is reported with error bars indicating the standard deviations (SD) of triplicate values. For *in vitro* phenotype, adherence, and growth assays, *n* = 3 to 12 for each strain and the mean ± SD is reported (except when representative images are shown). For the mouse systemic infection model, *n* = 6 for each strain, which has been shown to be sufficient for providing statistical power (24). The rat catheter model was performed in biological duplicate. This animal model provides a test of clinical relevance and is not a quantitative assessment of biofilm formation. Mice for the systemic infection model were randomized for survival and kidney burden measurements. Investigators were not blinded to the group allocation or when assessing the outcomes of the systemic infection and catheter models.

### Deposited data.

All raw spectrum (.RAW) files from the proteomic and MSP-MS experiments in this study are available at the ProteoSAFe resource (ftp://MSV000079564@massive.ucsd.edu/; username MSV000079564, password Candida).

## SUPPLEMENTAL MATERIAL

Movie S1  Time-lapse videos of biofilm formation by the wild-type (SN250) reference strain and the *sap5*Δ/Δ, *sap6*Δ/Δ, and *sap5/6*ΔΔ/ΔΔ deletion mutant strains in the BioFlux instrument over 12 h. Download Movie S1, MOV file, 13.8 MB

Data Set S1  MS data supporting the proteomic and MSP-MS results of this study. Download Data Set S1, XLSX file, 0.2 MB

Figure S1  Global substrate specificity profiles of protease activity in conditioned medium from wild-type *C. albicans* (SN425) grown under biofilm (A) and planktonic (B) conditions. iceLogo representations for 24-h conditioned medium at 20 µg/ml following 15, 60, and 240 min of incubation with the MSP-MS peptide library (*P* ≤ 0.05 for residues colored by physicochemical property; n is norleucine). Download Figure S1, TIF file, 5.5 MB

Figure S2  Activity-normalized comparison of cleavage specificity for wild-type *C. albicans* (SN425) grown under biofilm and planktonic conditions with MSP-MS time points and approximately the same number of cleavages (60 and 240 min, respectively). (A) iceLogo substrate specificity representations for 24-h biofilm and planktonic conditioned medium (*P* ≤ 0.05 for residues colored by physicochemical property). (B) Quantification of the total shared and unique cleavages for the biofilm and planktonic conditions. Of the 74 shared cleavage sites indicated here, 5 were recategorized for Fig. 3 and S5 because they were differentially sensitive to pepstatin in the biofilm and planktonic assays (and therefore could not be assigned to the Saps under both conditions). Three of these shared sequences (P4-P4′) were recategorized as “unassigned planktonic” (WPSnNKVG, XSAnnKIG, and TVNKQLRX), and two of these shared sequences (EVNDDVKX and GHVKLFRF) were recategorized as “unassigned biofilm.” (C) Heat map representation of biofilm and planktonic specificity differences with *Z* scores at the P4-P4′ positions. Biofilm-favored residues are blue (*Z* score, >0), and planktonic-favored residues are red (*Z* score, <0). Download Figure S2, TIF file, 4.5 MB

Figure S3  Global substrate specificity profiling of recombinantly produced Kex2 from *S. cerevisiae*. (A) Time-dependent generation of cleavages following dibasic (P2-P1) K/R-R residues in the MSP-MS library for recombinant Kex2 and *C. albicans* wild-type (SN425) conditioned medium from 24-h biofilm and planktonic cultures. Cleavage sites assigned to Kex2 in the conditioned medium profiles were sensitive to EDTA and insensitive to pepstatin treatments. (B) Kex2 displays MSP-MS cleavage sites distinct from those of Sap5 and Sap6. This is illustrated in the differential cleavage pattern of an example MSP-MS peptide. Download Figure S3, TIF file, 2.5 MB

Figure S4  Comparison of Sap5 and Sap6 global substrate specificity profiles. (A) Quantification of the total shared and unique cleavages for Sap5 and Sap6 at the 240-min MSP-MS time point. (B) Heat map representation of Sap5 and Sap6 specificity differences at the 240-min time point calculated by using *Z* score differences at the P4-P4′ positions. Sap5-favored residues are blue (*Z* score, >0), and Sap6-favored residues are red (*Z* score, <0). Download Figure S4, TIF file, 1.9 MB

Figure S5  Global biofilm and planktonic substrate specificity profiles for cleavages not assignable to Sap5 or Sap6. (A) iceLogo representations of biofilm condition-unique (*n* = 49), planktonic-condition-unique (*n* = 112), and shared (*n* = 55) cleavages by using activity-matched MSP-MS time points (*P* ≤ 0.05 for residues colored by physicochemical property). (B) Distribution of cleavage sites along the 14-mer peptide substrates. The peptides in the library are uncapped, allowing for the measurement of both exo- and endopeptidase activities. Planktonic-unique cleavages not assignable to Sap5 or Sap6 have an enrichment of aminopeptidase-like specificity compared to unassigned shared and unassigned biofilm-unique cleavages. Sap5 and Sap6 activities display predominant endopeptidase-like specificity. Download Figure S5, TIF file, 8.1 MB

Figure S6  Cleavage time courses for recombinantly produced Sap5 (closed circles) and Sap6 (open circles) against a 25-member sublibrary of MSP-MS peptide substrates. Spectral counts are plotted at 1, 5, 15, 30, 120, 240, and 1,440 min. Cleavage products are separated by preference for Sap5 (A), both Saps (B), or Sap6 (C). Duplicated peptides are cleaved at distinct sites. Although included in the sublibrary, the peptide HIGLQVHnRYINVn is not shown because of inconsistent time-dependent spectral count data. Download Figure S6, TIF file, 11.5 MB

Figure S7  Evaluation of fluorogenic substrate selectivity. Activity against recombinant Sap5 and Sap6 (A) and 24-h conditioned medium from wild-type *C. albicans* (SN425 and SN250 strains) grown under biofilm and planktonic conditions (B) was assayed. Aspartyl protease activity in conditioned medium was confirmed through pretreatment with 10 µM pepstatin A. For all results, mean activity is reported with error bars indicating the SD of triplicate values. Download Figure S7, TIF file, 4.9 MB

Figure S8  *SAP5/6* Deletion does not compromise *C. albicans* growth *in vitro* under planktonic conditions or *C. albicans* virulence *in vivo* in a planktonic model of hematogenously disseminated candidiasis. (A) Comparison of the growth rates under planktonic conditions of the wild-type (WT) reference *C. albicans* strain (SN250), the *sap5*Δ/Δ and *sap6*Δ/Δ deletion strains, and the corresponding *sap5*/*SAP5* and *sap6/SAP6* complemented strains illustrating no significant difference (n.s.) in planktonic growth rates compared to the wild-type reference (*P* ≥ 0.60) with *n* = 6 for each strain. Error bars indicate standard errors. Data were recorded every 15 min but are presented every 120 min for clarity. (B) In the mouse model of hematogenously disseminated candidiasis, female BALB/c mice underwent infection with the *sap5/6*ΔΔ/ΔΔ deletion mutant strain (5.4 × 10^5^ cells) or the wild-type (SN250) reference strain (5.3 × 10^5^ cells) with *n* = 6 mice per group. (B, left) Survival curves demonstrating no significant difference (*P* = 0.35) between the groups in the time to death. Statistical significance was calculated with a log-rank test. The survival times (reported as mean ± SD) were 1.7 ± 0.5 and 2.0 ± 0.9 days for mice infected with the wild-type and *sap5/6*ΔΔ/ΔΔ deletion mutant strains, respectively. (B, right) Box-and-whisker plot demonstrating no significant difference between the kidney burdens (*P* = 0.81) of the groups. Download Figure S8, TIF file, 6.9 MB
