# Supplementary figures and images for: Global Identification of Biofilm-Specific Proteolysis in Candida albicans
Source: mBio. 2016 Sep 13;7(5):e01514-16. doi: 10.1128/mBio.01514-16 (PMC5021810; doi:10.1128/mBio.01514-16)

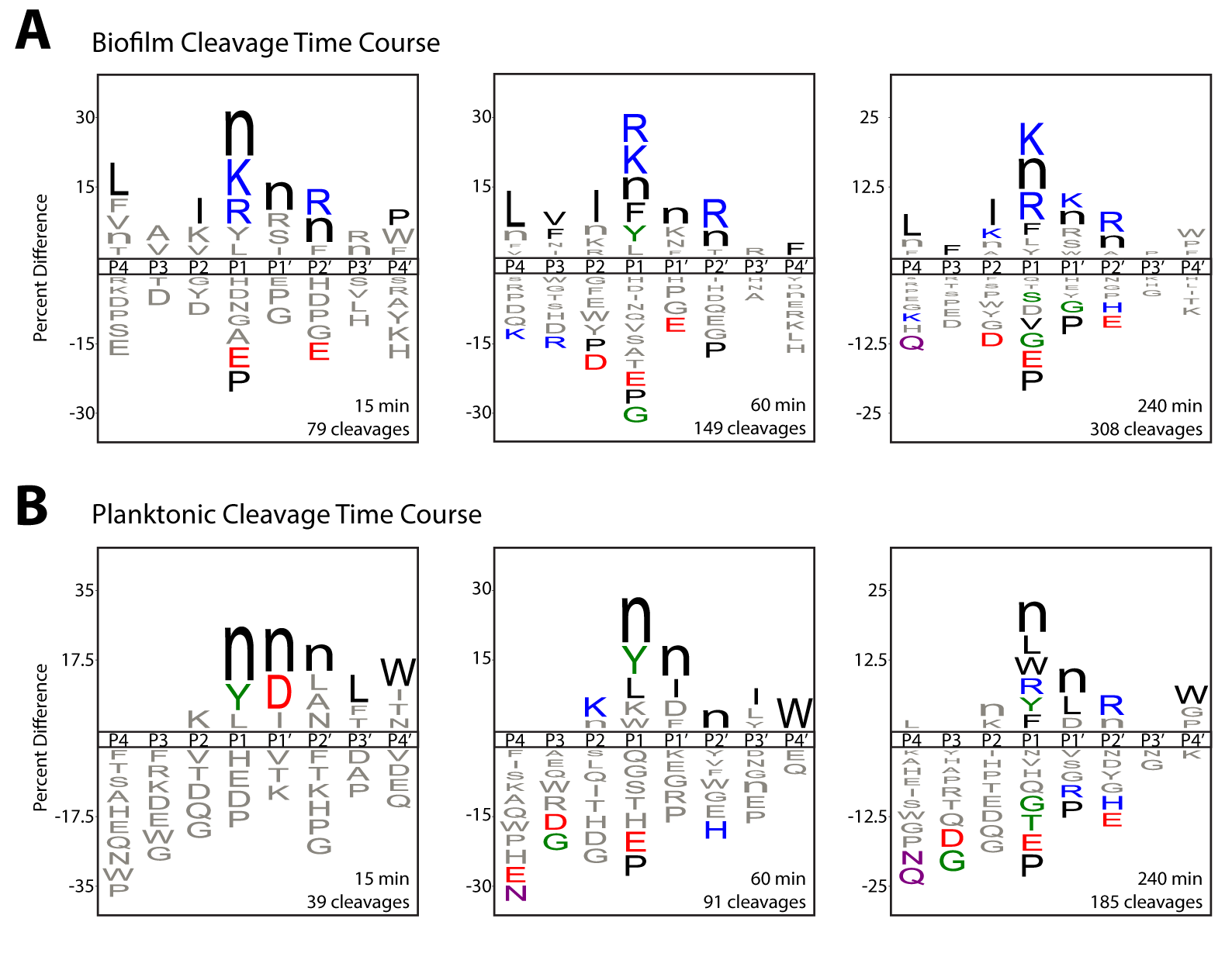

Supplement: Figure S1 — Global substrate specificity profiles of protease activity in conditioned medium from wild-type C. albicans (SN425) grown under biofilm (A) and planktonic (B) conditions. iceLogo representations for 24-h conditioned medium at 20 µg/ml following 15, 60, and 240 min of incubation with the MSP-MS peptide library (P ≤ 0.05 for residues colored by physicochemical property; n is norleucine). Download [file mbo004162993sf1.tif]

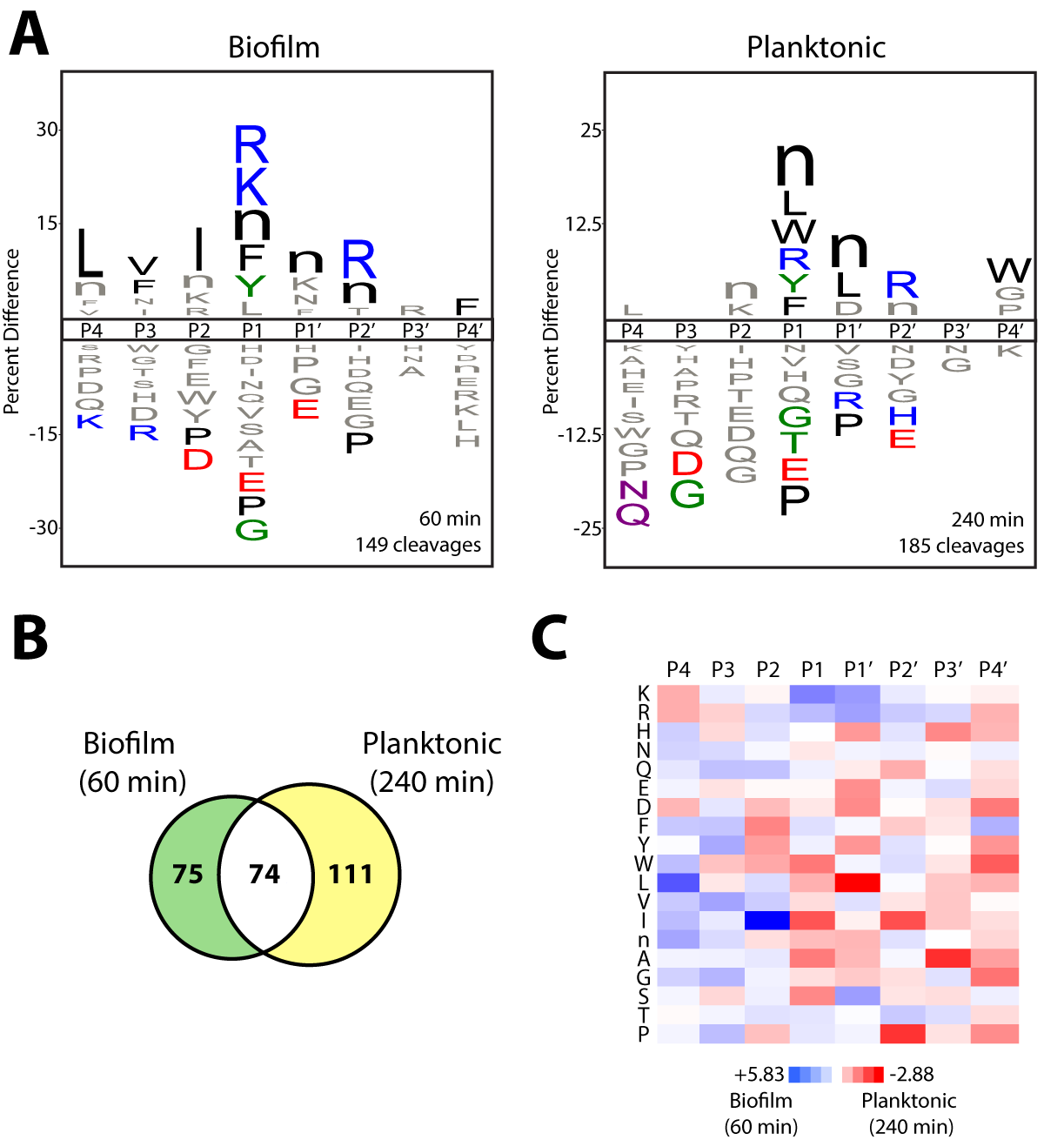

Supplement: Figure S2 — Activity-normalized comparison of cleavage specificity for wild-type C. albicans (SN425) grown under biofilm and planktonic conditions with MSP-MS time points and approximately the same number of cleavages (60 and 240 min, respectively). (A) iceLogo substrate specificity representations for 24-h biofilm and planktonic conditioned medium (P ≤ 0.05 for residues colored by physicochemical property). (B) Quantification of the total shared and unique cleavages for the biofilm and planktonic conditions. Of the 74 shared cleavage sites indicated here, 5 were recategorized for Fig. 3 and S5 because they were differentially sensitive to pepstatin in the biofilm and planktonic assays (and therefore could not be assigned to the Saps under both conditions). Three of these shared sequences (P4-P4′) were recategorized as “unassigned planktonic” (WPSnNKVG, XSAnnKIG, and TVNKQLRX), and two of these shared sequences (EVNDDVKX and GHVKLFRF) were recategorized as “unassigned biofilm.” (C) Heat map representation of biofilm and planktonic specificity differences with Z scores at the P4-P4′ positions. Biofilm-favored residues are blue (Z score, >0), and planktonic-favored residues are red (Z score, <0). Download [file mbo004162993sf2.tif]

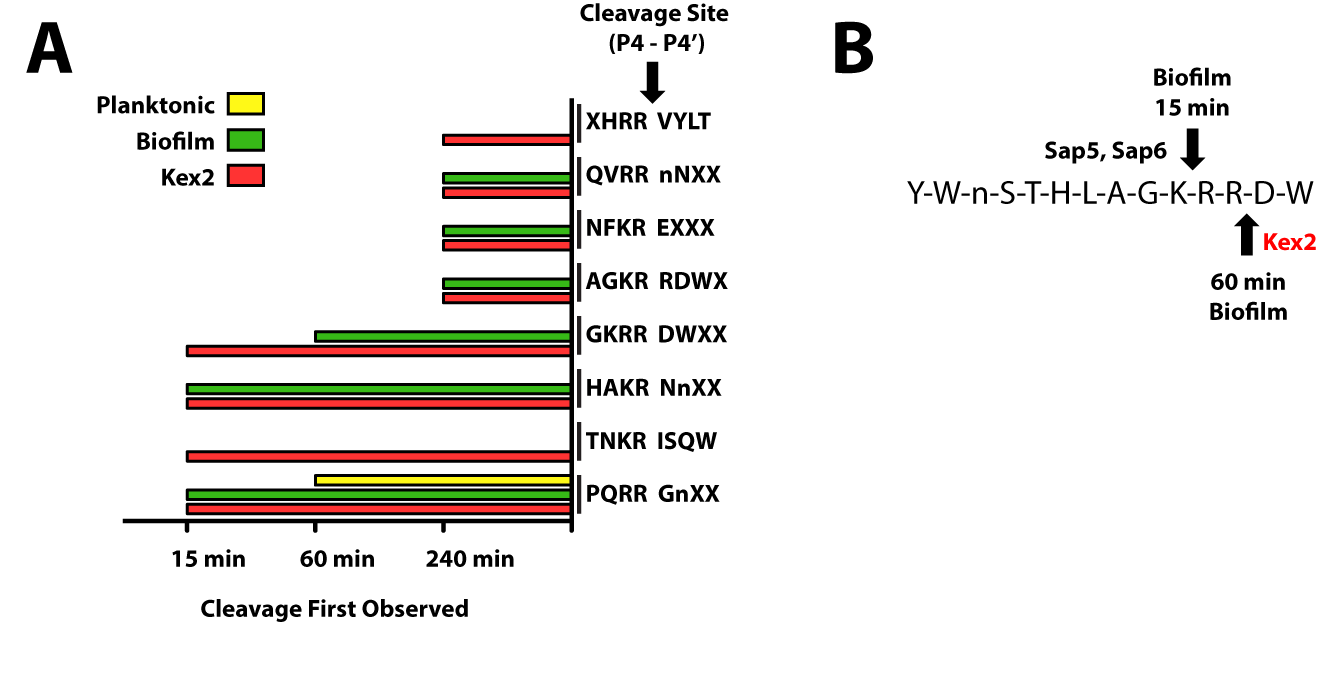

Supplement: Figure S3 — Global substrate specificity profiling of recombinantly produced Kex2 from S. cerevisiae. (A) Time-dependent generation of cleavages following dibasic (P2-P1) K/R-R residues in the MSP-MS library for recombinant Kex2 and C. albicans wild-type (SN425) conditioned medium from 24-h biofilm and planktonic cultures. Cleavage sites assigned to Kex2 in the conditioned medium profiles were sensitive to EDTA and insensitive to pepstatin treatments. (B) Kex2 displays MSP-MS cleavage sites distinct from those of Sap5 and Sap6. This is illustrated in the differential cleavage pattern of an example MSP-MS peptide. Download [file mbo004162993sf3.tif]

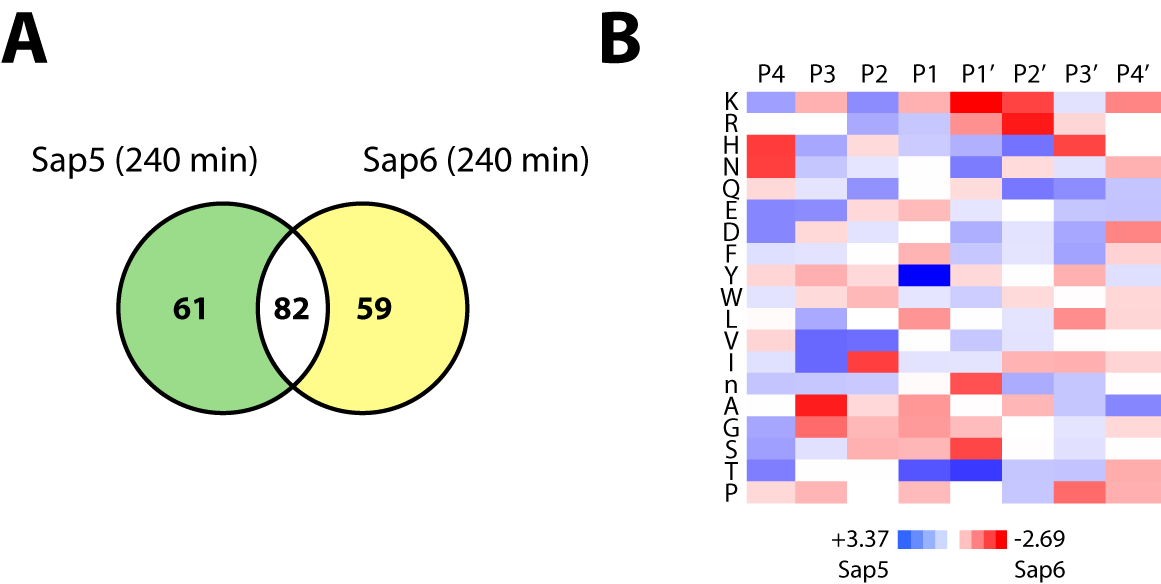

Supplement: Figure S4 — Comparison of Sap5 and Sap6 global substrate specificity profiles. (A) Quantification of the total shared and unique cleavages for Sap5 and Sap6 at the 240-min MSP-MS time point. (B) Heat map representation of Sap5 and Sap6 specificity differences at the 240-min time point calculated by using Z score differences at the P4-P4′ positions. Sap5-favored residues are blue (Z score, >0), and Sap6-favored residues are red (Z score, <0). Download [file mbo004162993sf4.tif]

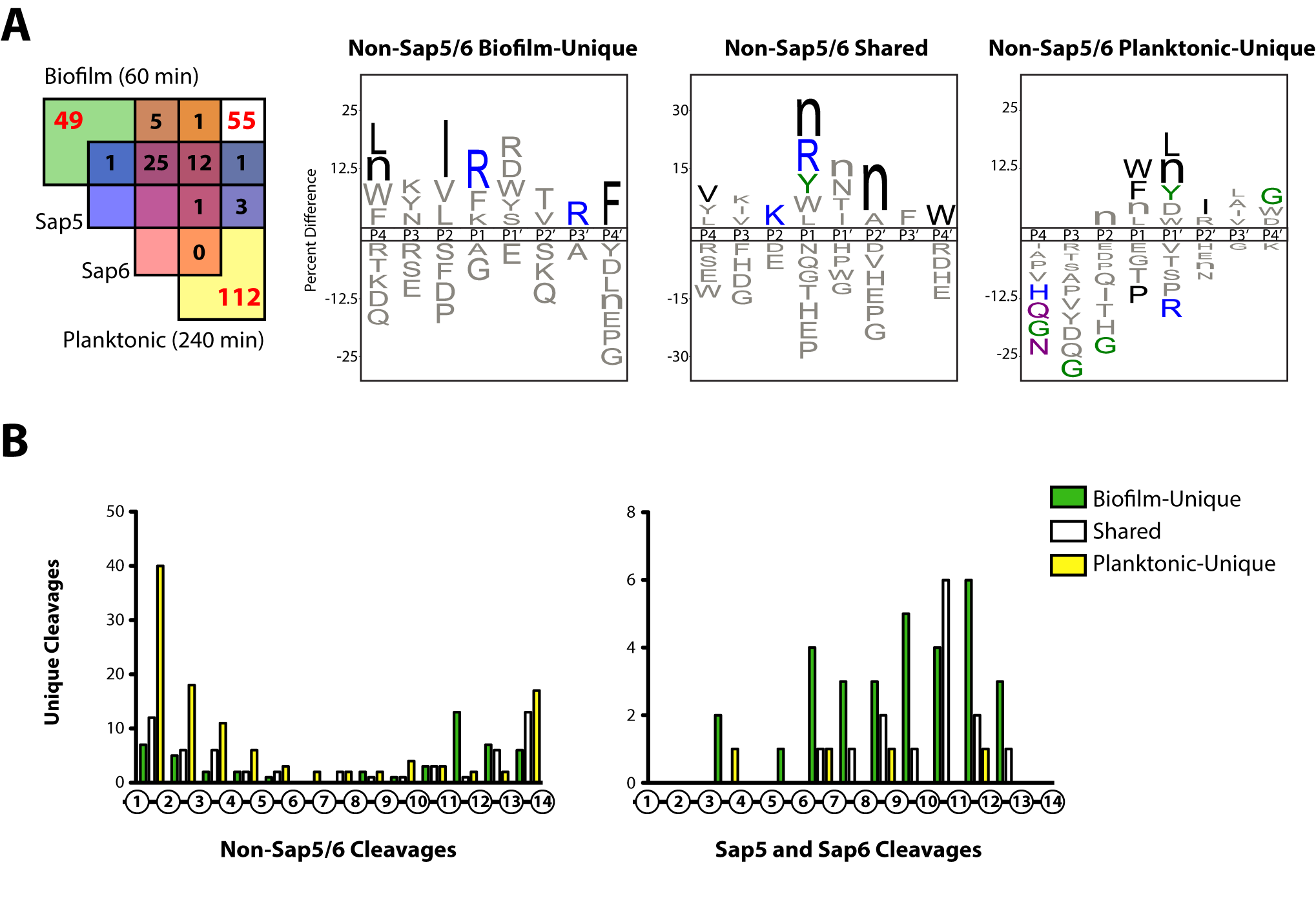

Supplement: Figure S5 — Global biofilm and planktonic substrate specificity profiles for cleavages not assignable to Sap5 or Sap6. (A) iceLogo representations of biofilm condition-unique (n = 49), planktonic-condition-unique (n = 112), and shared (n = 55) cleavages by using activity-matched MSP-MS time points (P ≤ 0.05 for residues colored by physicochemical property). (B) Distribution of cleavage sites along the 14-mer peptide substrates. The peptides in the library are uncapped, allowing for the measurement of both exo- and endopeptidase activities. Planktonic-unique cleavages not assignable to Sap5 or Sap6 have an enrichment of aminopeptidase-like specificity compared to unassigned shared and unassigned biofilm-unique cleavages. Sap5 and Sap6 activities display predominant endopeptidase-like specificity. Download [file mbo004162993sf5.tif]

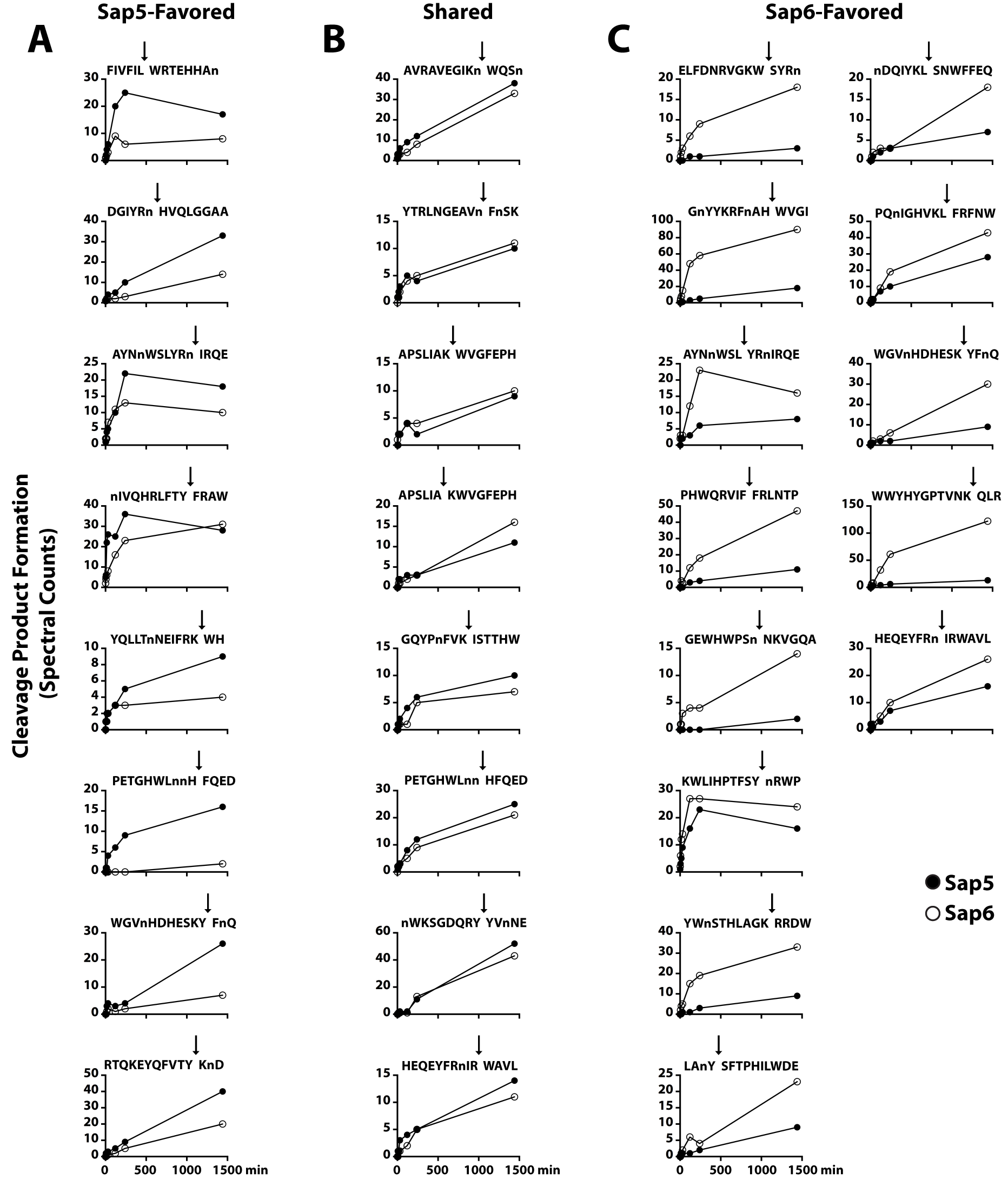

Supplement: Figure S6 — Cleavage time courses for recombinantly produced Sap5 (closed circles) and Sap6 (open circles) against a 25-member sublibrary of MSP-MS peptide substrates. Spectral counts are plotted at 1, 5, 15, 30, 120, 240, and 1,440 min. Cleavage products are separated by preference for Sap5 (A), both Saps (B), or Sap6 (C). Duplicated peptides are cleaved at distinct sites. Although included in the sublibrary, the peptide HIGLQVHnRYINVn is not shown because of inconsistent time-dependent spectral count data. Download [file mbo004162993sf6.tif]

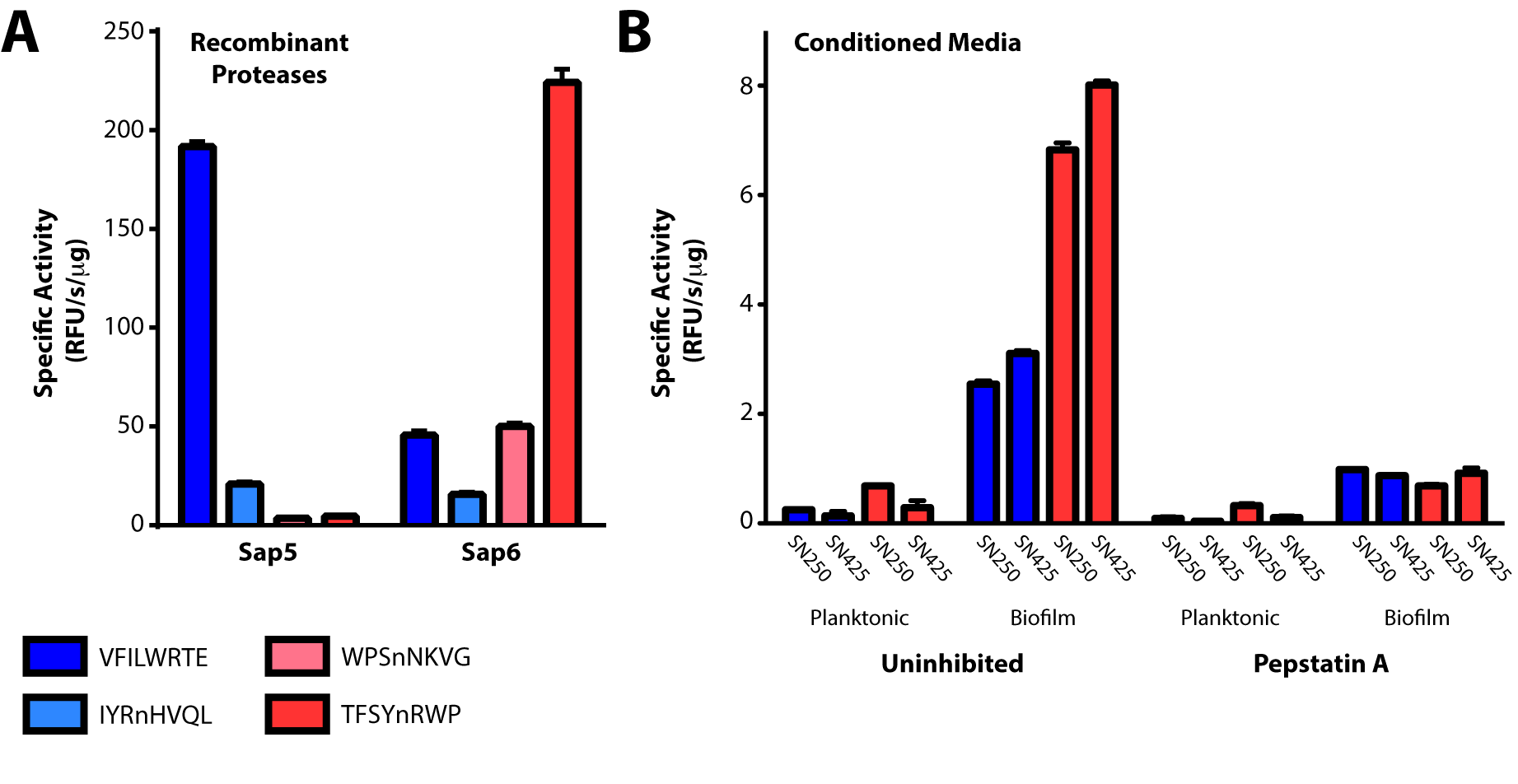

Supplement: Figure S7 — Evaluation of fluorogenic substrate selectivity. Activity against recombinant Sap5 and Sap6 (A) and 24-h conditioned medium from wild-type C. albicans (SN425 and SN250 strains) grown under biofilm and planktonic conditions (B) was assayed. Aspartyl protease activity in conditioned medium was confirmed through pretreatment with 10 µM pepstatin A. For all results, mean activity is reported with error bars indicating the SD of triplicate values. Download [file mbo004162993sf7.tif]

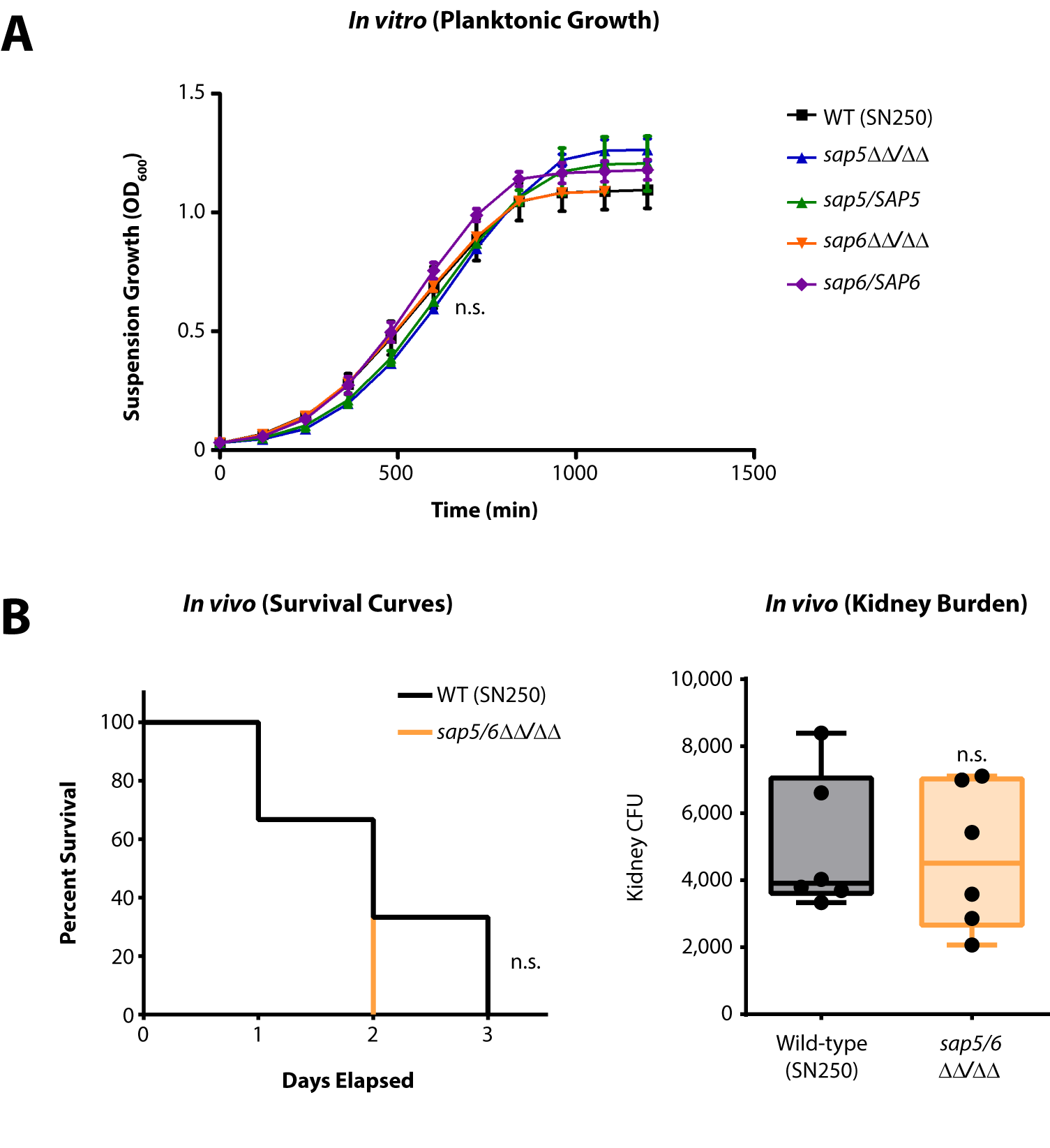

Supplement: Figure S8 — SAP5/6 Deletion does not compromise C. albicans growth in vitro under planktonic conditions or C. albicans virulence in vivo in a planktonic model of hematogenously disseminated candidiasis. (A) Comparison of the growth rates under planktonic conditions of the wild-type (WT) reference C. albicans strain (SN250), the sap5Δ/Δ and sap6Δ/Δ deletion strains, and the corresponding sap5/SAP5 and sap6/SAP6 complemented strains illustrating no significant difference (n.s.) in planktonic growth rates compared to the wild-type reference (P ≥ 0.60) with n = 6 for each strain. Error bars indicate standard errors. Data were recorded every 15 min but are presented every 120 min for clarity. (B) In the mouse model of hematogenously disseminated candidiasis, female BALB/c mice underwent infection with the sap5/6ΔΔ/ΔΔ deletion mutant strain (5.4 × 10^5^ cells) or the wild-type (SN250) reference strain (5.3 × 10^5^ cells) with n = 6 mice per group. (B, left) Survival curves demonstrating no significant difference (P = 0.35) between the groups in the time to death. Statistical significance was calculated with a log-rank test. The survival times (reported as mean ± SD) were 1.7 ± 0.5 and 2.0 ± 0.9 days for mice infected with the wild-type and sap5/6ΔΔ/ΔΔ deletion mutant strains, respectively. (B, right) Box-and-whisker plot demonstrating no significant difference between the kidney burdens (P = 0.81) of the groups. Download [file mbo004162993sf8.tif]
